# Decomposition-driven biomarker identification enhances non-invasive early cancer detection

**DOI:** 10.1016/j.isci.2026.117621

**Published:** 2026-09-24

**Authors:** Weizhong Chen, Ying-Hong An, Rui-Qing Fu, Hai-Xiang Sun, Wen-Jing Zheng, Fang Wang, Hua Chen, Wei Li, Min-Jie Xu, Cheng-Cheng Ma, Zhi-Xi Su, Rui Liu, Xin-Rong Yang

**Affiliations:** 1 Precision Medicine Center, Zhongshan Hospital, Fudan University, Shanghai 200032, China; 2 Institute of Clinical Science, Zhongshan Hospital, Fudan University, Shanghai 200032, China; 3 Department of Hepatobiliary Surgery and Liver Transplantation, Liver Cancer Institute, Zhongshan Hospital, and Key Laboratory of Carcinogenesis and Cancer Invasion of Ministry of Education, Fudan University, Shanghai 200032, China; 4 Department of Clinical Laboratory, Air Force Medical Center, Air Force Medical University, Beijing 100142, China; 5 Singlera Genomics (Shanghai) Ltd., Shanghai 201203, China; 6 Department of Gastroenterology and Hepatology, Tianjin Third Central Hospital, 83 Jintang Road, Hedong, Tianjin 300170, China

**Keywords:** marker selection, data decomposition, early cancer detection, diagnostics, biomarker, TOO marker

## Abstract

Accurate marker selection in cell-free DNA (cfDNA) is essential for non-invasive early cancer detection, yet conventional approaches based on bulk tissue data often yield markers with limited efficacy in blood-based assays, due to the sparse and heterogeneous nature of tumor-derived cfDNA released into the bloodstream during early tumorigenesis. Here, we present a component decomposition framework that leverages non-negative matrix factorization (NMF) to resolve distinct methylation components from bulk reduced representation bisulfite sequencing (RRBS) data of five gastrointestinal cancers and their para-carcinoma tissues. This approach demonstrates that cancer-specific signals can be recovered at the component level, enabling the identification of cancer-specific and tissue-of-origin markers with significantly improved performance in cfDNA. Extending this framework to transcriptomic data further validates its utility for cfRNA-based biomarker discovery and molecular subtyping. Together, our framework offers a robust and scalable solution to improve biomarker identification for early cancer detection and patient stratification.

## Highlights

• NMF decomposition resolves heterogeneous methylation signals in tumors
• Decomposition improves cancer biomarker selection from bulk tissue
• NMF-derived markers improve prediction in independent cfDNA data
• Component decomposition improves tissue-of-origin marker prioritization

## Graphical abstract

**Figure:**
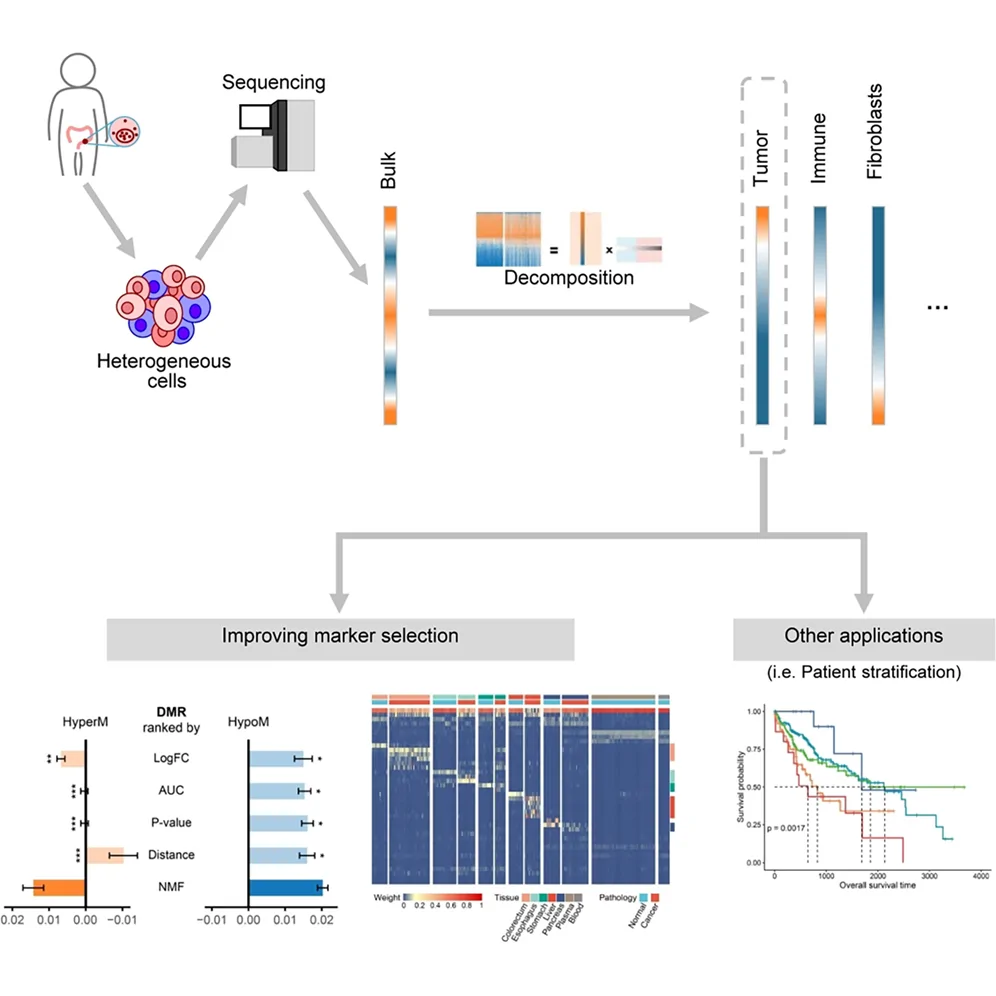

## Teaser

Biomarker; Diagnostics; Epigenetics

## Introduction

Cancer early detection aims to identify cancer-associated molecular signals at precancerous lesions or early clinical stages (primarily stage I–II) prior to definitive diagnosis, enabling applications in screening, early diagnosis, and surveillance across average-risk, symptomatic, and epidemiologically defined high-risk populations. Blood-based non-invasive diagnostic approaches have emerged as a promising strategy for early cancer detection.^1^ Among those, methylation-based assays, particularly those targeting specific markers in circulating cell-free DNA (cfDNA), have demonstrated substantial clinical potential.^2^^,^^3^^,^^4^^,^^5^ A pivotal step in developing such assays is the robust selection of differentially methylated markers capable of distinguishing cancer from non-cancer states. This selection process typically involves two key stages. The first is the discovery stage, in which differentially methylated CpG sites (DMCs) or differentially methylated regions (DMRs) are identified as candidate markers through comparative analyses between cancer tissues and matched para-carcinoma or normal tissues. The second is the validation stage, where the candidate markers are assessed for their ability to discriminate cancer against non-cancer samples in plasma cfDNA. While the discovery stage establishes the potential association of candidate markers with cancer signals at the tissue level, the subsequent validation stage ensures that these signals are retained and detectable in the blood-based context. Ultimately, validated markers are assembled into a targeted panel optimized for non-invasive cancer detection.^6^

However, two major challenges in the discovery and validation stages hinder the identification of high-quality cfDNA methylation markers, often leading to the poor performance of tissue-derived candidates in blood-based evaluation. In the discovery stage, marker selection is confounded by the pronounced heterogeneity of tumor tissues,^7^^,^^8^ which consist not only of malignant cells but also of a complex microenvironment, including blood vessels and diverse infiltrating immune cells.^9^^,^^10^^,^^11^ Consequently, many candidate markers may capture signals from non-malignant components rather than true cancer-specific alterations, thereby limiting their specificity and generalizability. For instance, increased immune cell infiltration can lead to the selection of markers that reflect inflammation or immune response, rather tumor-intrinsic feature. In the validation stage, a high level of sensitivity is required due to the low abundance of circulating tumor DNA (ctDNA) in plasma, especially in early-stage cancers.^12^ Cancer-associated methylation signals originating from tumor tissue are often substantially diluted upon release into the bloodstream. Moreover, background noise from hematopoietic cells, such as white blood cells (WBCs), can mask or mimic cancer-specific methylation patterns. For example, a hyper-methylated marker identified in tumor tissue may lack discriminatory power in plasma if the same locus is also hyper-methylated in WBCs. Taken together, these challenges suggest that identifying methylation markers from purified cancer components within tumor tissues, coupled with filtering of background noise, would improve the performance of markers in cfDNA-based assays for early, non-invasive cancer detection.

Single-cell or cell sorting-based methylation sequencing offers high-resolution approaches for identifying cancer-specific methylation markers,^13^^,^^14^ but their widespread application is currently limited by high costs and technical complexity. Alternatively, the deconvolution of bulk methylation sequencing data provides a cost-effective and scalable solution for investigating tissue composition and is readily applicable to the vast repositories of existing bulk methylation datasets. Several deconvolution approaches, such as MethylCIBERSORT,^15^ MethylResolver,^16^ and HiTIMED,^17^ have been developed based on the regression of bulk data against the reference methylation profiles of known cell types. Although these reference-based methods offer high accuracy, they are inherently limited by the availability and completeness of appropriate reference data, which is often lacking. To overcome this limitation, reference-free approaches like RefFreeEWAS^18^ and MeDeCom^19^ have been developed. These methods leverage non-negative matrix factorization (NMF) to decompose the observed data into a set of latent components. These components have been shown to correspond to underlying data phenotype^18^ and to reflect major cell types within heterogeneous tissues.^19^ However, despite growing interest in uncovering these latent components, few studies have systematically evaluated the utility of the decomposed data for methylation marker discovery.

In this study, we hypothesized that methylation signals within tissues are highly heterogeneous, and that decomposition of bulk methylation data can deconvolute these averaged signals to reveal components from distinct cellular or tissue sources. We further hypothesized that markers derived from tumor-specific components are more tumor-specific, and thus are expected to produce stronger and more reliable signals in plasma cfDNA, enhancing the potential for non-invasive early cancer detection. To address these hypotheses, we propose a novel framework to identify differential methylation markers associated with sub-tissue-specific components, leveraging an unsupervised NMF approach selected for its adaptability and interpretability (Figure 1A). The bulk RRBS (reduced representation bisulfite sequencing, profiling DNA methylation in CpG-rich regions rather than the entire genome) data from five gastrointestinal (GI) cancers and their para-carcinoma tissues were subjected to decomposition. We analyzed methylation differences at the component level, compared marker selection outcomes between our method and conventional bulk-based approaches, and further extended our method to identify cancer type-specific markers for tissue-of-origin (TOO) prediction, which is typically performed in multi-cancer early detection (MCED) to determine the specific cancer type in positive cases. Additionally, we demonstrated the broader applicability of our framework to other biomarker types, such as cell-free RNA (cfRNA), highlighting its utility not only in marker discovery but also in potential cancer subtype classification. Overall, our results demonstrated that decomposition-based analysis significantly enhances marker identification, suggesting that this method could inform the next generation of marker selection strategies for non-invasive cancer diagnostics. Importantly, the current study is centered on the development and assessment of a decomposition-based biomarker selection strategy. While the candidate markers were evaluated against an external public cfDNA dataset, they have not been subjected to comprehensive experimental validation using a dedicated methylation panel in a large-scale independent clinical cohort. Such validation represents an essential prerequisite for advancing the proposed framework toward routine clinical application.

**Figure 1:**
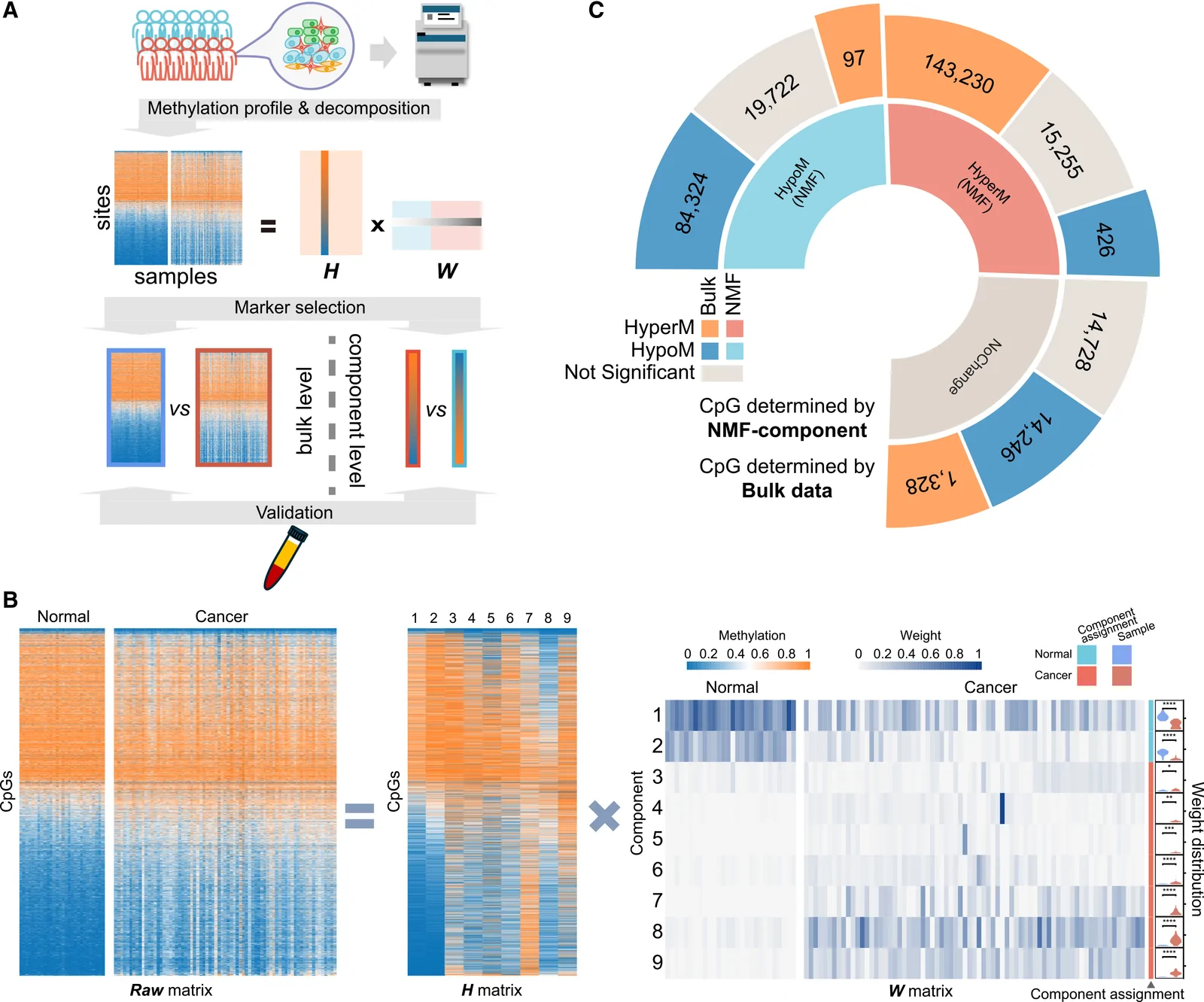
Decomposition of bulk DNA methylation data (A) An illustration showing the decomposition of bulk sequencing data from participants with different conditions (e.g., cancer and normal) and comparisons between the markers identified at the bulk and component levels. CfDNA samples were used to validate the selected markers. (B) The decomposition of colorectal cancer (CRC) and para-carcinoma RRBS data resulted in nine components, represented as the product of the *H* matrix and the *W* matrix. Each component was assigned to either a normal or cancer condition, according to the distribution of component weights across the samples (by *t* test). (C) Comparison of CpG alteration patterns between CRC and normal samples before and after decomposition. CpGs were defined as hyper-methylated (HyperM) or hypo-methylated (HypoM) CpGs in bulk data if they were significantly hyper-methylated or hypo-methylated in cancer samples (*p* < 0.05; *U* test followed by Benjamini-Hochberg correction), and were defined as HyperM or HypoM CpGs if their log_2_(fold-change) was >0.1 or < −0.1 between the average methylation of cancer-associated components and that of normal-associated components. The numbers (labeled in the plot) were log_10_-scaled for display. Significance: ∗*p* < 0.05, ∗∗*p* < 0.01, ∗∗∗*p* < 0.001, ∗∗∗∗*p* < 0.0001. See also Figures S1–S4.

## Results

### Discrepancies between methylation markers at the bulk and component levels

Bulk RRBS data for 199 samples from 73 patients with colorectal cancer (CRC), 31 with esophageal cancer, 19 with stomach cancer, 28 with liver cancer, and 48 with pancreatic cancer, and 153 para-carcinoma samples (28 from patients with colorectal cancer, 42 with esophageal cancer, 27 with stomach cancer, 26 with liver cancer, and 30 with pancreatic cancer) were collected for analysis.^6^

Tumor tissues are heterogeneous mixtures of multiple cellular and sub-tissue components, resulting in composite bulk methylation signals. To model this heterogeneity, we applied non-negative matrix factorization (NMF) to decompose the bulk methylation matrix into a methylation spectrum matrix and a weight fraction matrix, representing component-specific methylation patterns and their relative contributions across samples. Mathematically, for each cancer type, CpG methylation values for both para-carcinoma (normal) and cancer samples were combined into a single matrix (with *m* CpG sites in the rows and *n* samples in the columns), which was then decomposed into two matrices: the methylation matrix (i.e., *H* matrix, with *m* CpG sites × *K* components) and the weight matrix (i.e., *W* matrix, with *K* components × *n* samples). The *H* matrix depicted the methylation profiles across CpGs for each component, while the *W* matrix quantified the weights, which can be normalized as the fractions of the components in each sample (Figures 1B and S1A–S4A; STAR Methods). Thus, the observed methylation profile of bulk data could be treated as the result of a mixture of different tissue components with different fractions. As a consequence, it shed light on the composition of bulk methylation signals and revealed the methylation alterations at a higher resolution. Next, we use the data for colorectal samples to illustrate the decomposition of bulk data to investigate alterations in methylation between cancer and normal samples. Similar results were observed for all five cancer types (see supplemental information for other cancer types).

Compared with the raw matrix, the CpGs in the *H* matrix showed distinct methylation patterns across different components. Notably, components exhibited clearly different enrichments in either normal samples or cancer samples (Figure 1B), indicating associations between components and different sample pathological conditions. We then examined the consistency of methylation-altered CpGs before and after decomposition (Figures 1C and S1B–S4B). The CpGs were defined as hyper-methylated (HyperM) or hypo-methylated (HypoM) in cancers by comparing their average methylation levels between cancer and normal samples (before decomposition), or comparing between the average methylation signal (in the *H* matrix) across cancer- and that across normal-associated components (after decomposition) (e.g., components 3–9 and 1–2 in Figure 1B, which showed significantly enriched fractions in cancer and normal samples, respectively, were defined as cancer- and normal-associated components; STAR Methods). A substantial proportion of CpGs retained their methylation change patterns before and after decomposition (85.2% of bulk HypoM, 99.0% of bulk HyperM, and 29.6% of bulk “no-change” CpGs). However, 17.4% CpGs exhibited altered methylation patterns following decomposition (14.4% HypoM and 0.92% HyperM reclassified as “no-change” CpGs; 39.7%/30.7% “no-change” CpGs reclassified as HypoM/HyperM, respectively), with 0.18% CpGs even showing reversed methylation trends (0.43% HypoM reclassified as HyperM CpGs; 0.067% HyperM reclassified as HypoM CpGs), reflecting discrepancies between differential CpGs identified at the tissue level and those identified at the component level. In particular, 70.4% of bulk “no-change” CpGs were identified as HypoM/HyperM CpGs at the component level, suggesting that conventional selection methods may overlook potentially informative markers that are detectable in blood. Conversely, 15.3% bulk HypoM/HyperM CpGs appeared to lose differential signal at the component level, indicating that these changes may arise from non-cancer components and thus lack true discriminative power between cancer and normal tissue. These observations implied that intrinsic methylation profiles of cellular components influenced the observed methylation patterns of bulk data, thereby impacting the identification of truly differential markers.

### Methylation signal of tissue markers is compromised by decomposed components

We next investigated the influence of the component-specific methylation profiles and fractions on tissue markers identified from bulk data. We first used bulk data to identify HyperM and HypoM markers for cancer by comparing cancer and normal samples in a conventional manner (e.g., group-wise comparison via *U* tests; STAR Methods),^3^^,^^20^ and then profiled their methylation spectra across decomposed components (Figures 2A and S5A–S8A). As anticipated, the methylation level of HypoM markers decreased from normal-associated components (components 1 and 2) to cancer-associated components (components 3–9), whereas the methylation level of HyperM markers increased along the same gradient. However, a subset of markers exhibited discordant patterns of methylation changes at the component level (Figures 2B and S5B–S8B), particularly among the bulk HypoM markers, where 43 out of 199 HypoM markers were conversely hyper-methylated in cancer-associated components. This result indicated that bulk-level methylation signals are shaped by various components, and the discrepancies may partly explain the failure of tissue markers in blood samples, as the differential patterns may change at the component level.

**Figure 2:**
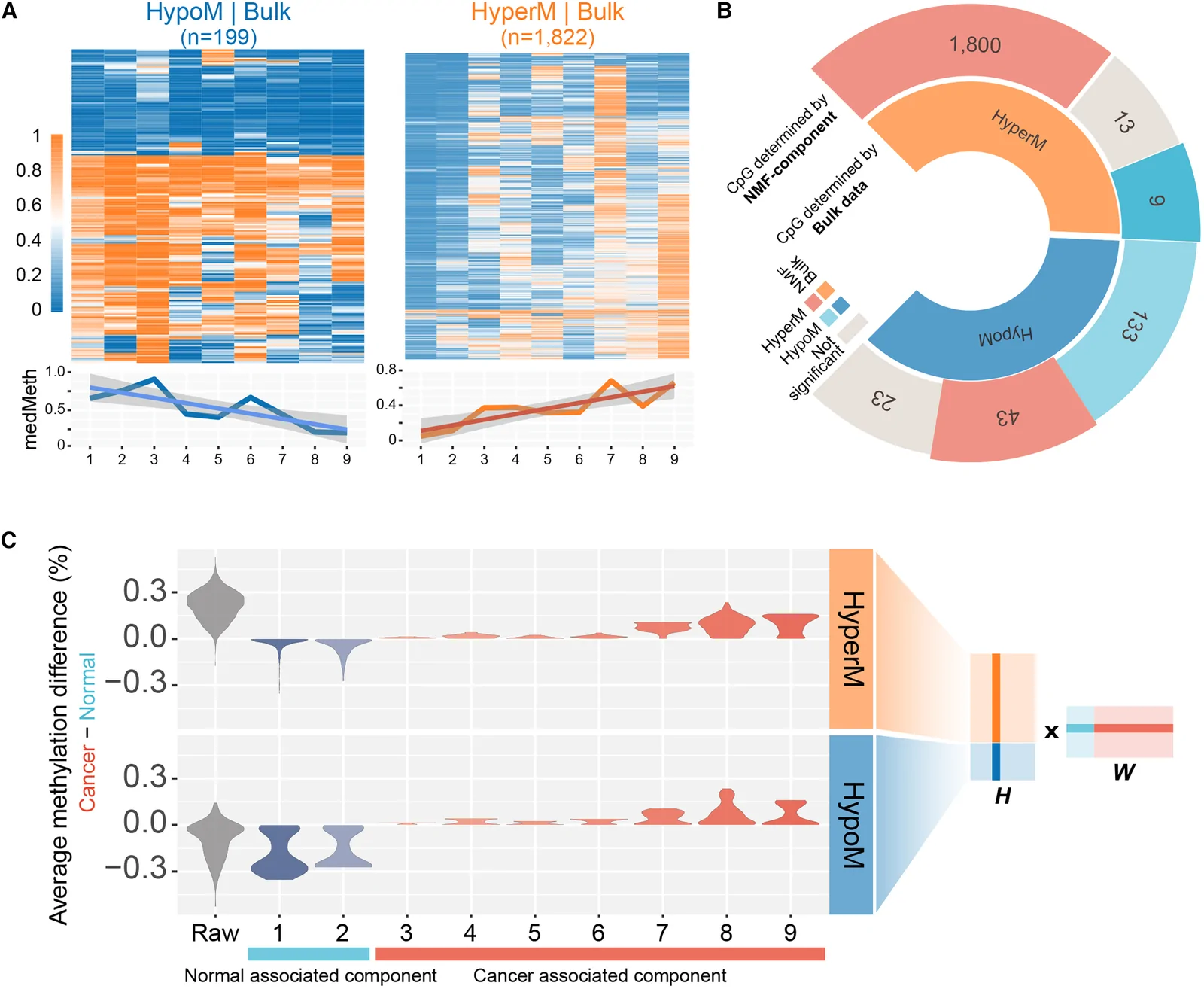
Contribution of different components to tissue markers (A) Methylation profiles of tissue cancer markers identified from RRBS CRC data across components, with median methylation values shown at the bottom. (B) Examination of methylation alteration patterns of tissue cancer markers at the component level. (C) The distribution of the average methylation differences between cancer and normal samples for HyperM and HypoM tissue markers, respectively, calculated using raw RRBS data, and each of the component spectra. To the right, the calculation of each component’s contribution was shown, where the methylation spectrum of a certain component was multiplied by its weights across samples, and average differences were calculated between cancer and normal samples. See also Figures S5–S8.

The decomposition allowed us to further quantitatively assess the contribution of each component to the bulk methylation signal, which can be approximated as the weighted sum of component-specific methylation levels by their respective fractions. For each HyperM and HypoM marker identified from bulk data, we calculated its weighted methylation across samples for each component and then examined the methylation alterations between cancer and normal groups. We found that the methylation differences of the bulk data-derived markers were primarily driven by the corresponding components, but were also attenuated by the opposite components (Figures 2C and S5C–S8C). For instance, all cancer-associated components (components 3–9) contributed to the increased methylation of HyperM markers in cancer samples, while the normal-associated components (components 1 and 2) modestly offset the difference. These findings highlight that the bulk cancer signal was a complex mixture of various components within the tissue, and the identification of tissue markers was based on a compromised signal.

### Blood samples validate the cancer signal of markers at the component level

We have demonstrated that the observed methylation profile was proportionally contributed by components with distinct methylation spectra, resulting in compromised marker signals. Hence, we hypothesized that a ‘pure’ cancer signal was produced by specific component(s), and that more robust blood-based cancer markers could be identified by contrasting such pure signals against background signals from hematopoietic cells, such as WBCs. To test this hypothesis, we first attempted to identify the component(s) most associated with cancer by correlating component-specific methylation with reference methylomes from a range of cell types, including hematopoietic cells (blood cells), macrophages, endothelial cells, tissue epithelial cells, and cancer cells (Figures 3A and S9A–S12A). For CRC, we found that components 2 and 8 were highly correlated with normal intestinal cells and CRC cell lines, respectively, while other components were associated with blood cells. Notably, over half of the normal and cancer samples exhibited substantial proportions (>0.2) of components 2 and 8, respectively. Based on these findings, we designated component 8 as the CRC-associated component, and thus the cancer signal in blood could be captured by comparing the methylation level of component 8 to that of WBCs.

**Figure 3:**
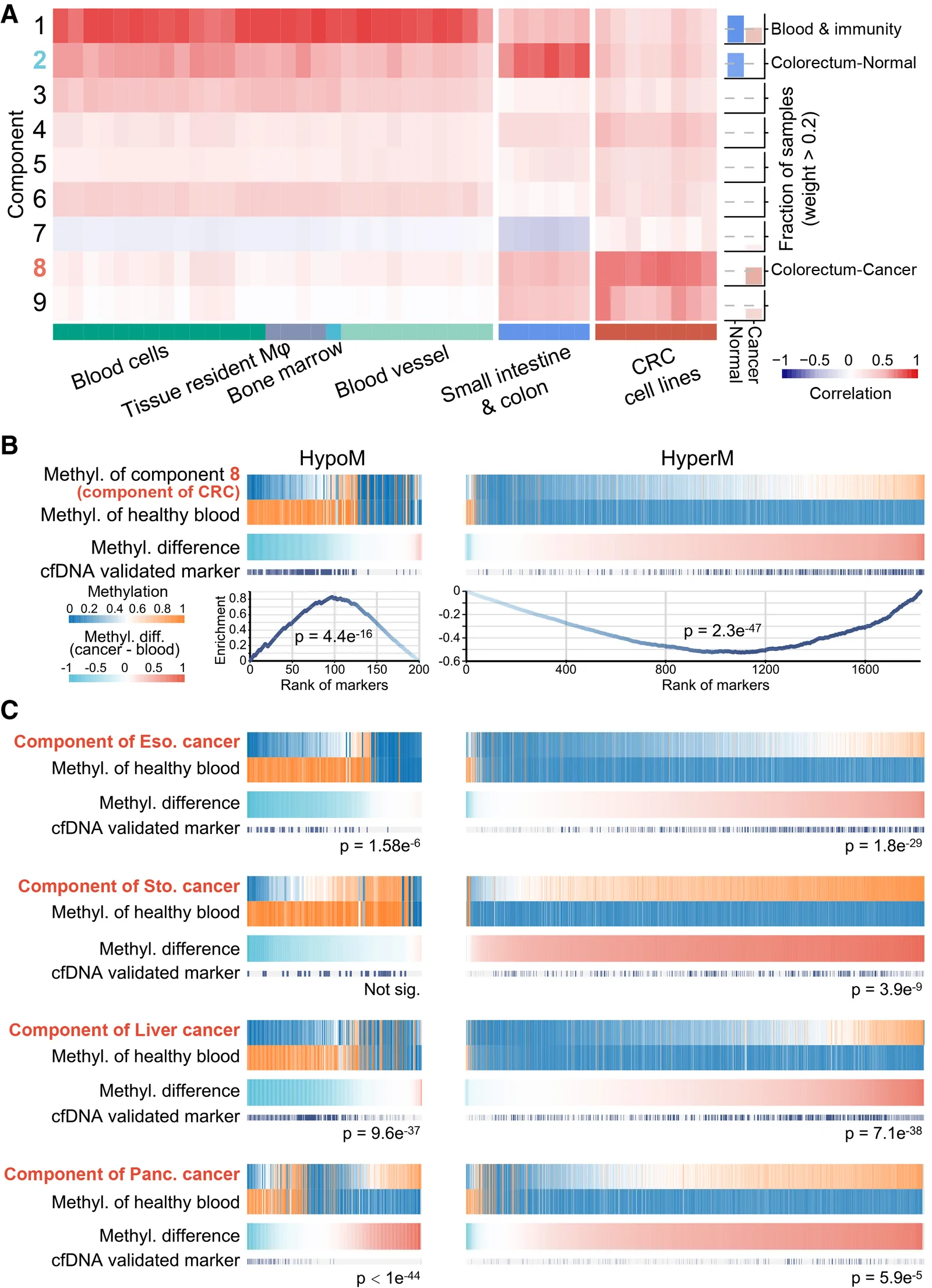
Component determination and validation of tissue cancer markers at the component level in cfDNA samples (A) The correlations between the methylation status of various cell types and CRC decomposed components, alongside the fraction of samples with a component weight >0.2 (on the right). Each column represents one cell type, and the major types are shown below. The most strongly associated tissue/cell types for certain components (e.g., components 2 and 8) were identified. The cell types evaluated include (from left to right): Blood-B, Blood-B-Mem, Blood-Granulocytes, Blood-NK, Blood-T-CD3, Blood-T-CD4, Blood-T-CenMem-CD4, Blood-T-EffMem-CD4, Blood-T-Naive-CD4, Blood-T-CD8, Blood-T-Eff-CD8, Blood-T-EffMem-CD8, Blood-T-Naive-CD8, Blood-Monocytes, Lung-Alveolar-Macrophages, Lung-interstitial-Macrophages, Colon-Macrophages, Liver-Macrophages, Bone marrow-Erythrocyte progenitors, Aorta-Endothelium, Saphenous-Vein-Endothelium, Lung-Alveolar-Endothelium, Liver-Endothelium, Pancreas-Endothelium, Pancreas-Islet-Endothelium, Kidney-Glomerular-Endothelium, Kidney-Tubular-Endothelium, Aorta-Smooth-Muscle, Coronary-Artery-Smooth-Muscle, Small-int-Endocrine, Small-int-Epithelium, Colon-Left-Endocrine, Colon-Left-Epithelium, Colon-Right-Endocrine, Colon-Right-Epithelium, HCC-56, HT55, LS-180, DIFI, NCI-H630, SNU-1040, SNU-175, and SNU-C2B. (B and C) The enrichment of cfDNA-validated markers (dark blue labeled) among previously defined HypoM and HyperM markers from an existing target methylation sequencing panel. The methylation spectra of the cancer-specific component and the average of healthy blood cell samples, and their differences are displayed. Markers were ranked by the methylation differences between the CRC-specific component and the average of healthy blood cell samples (B) and between the esophagus/stomach/liver/pancreas-specific component and the average of healthy blood cell samples (C). *p* values (<0.05) from the Kolmogorov-Smirnov test were labeled to indicate statistical significance. See also Figures S9–S13.

Next, we used an existing panel of target methylation sequencing for multiple cancer detection^6^ to validate the cancer signal associated with the cancer-specific component. For each target region of the more than one thousand pre-defined tissue-derived markers from the panel, we calculated the methylation difference between component 8 of CRC data and WBC RRBS data from 20 healthy samples, and ranked them based on their differences. Plasma cfDNA from a total of 845 cancer patients (200 colorectal, 150 esophageal, 159 stomach, 293 liver, and 43 pancreatic) and 832 healthy participants was also tested using the same sequencing panel. cfDNA-validated markers were determined whether they exhibited significant differences between cancer and normal cfDNA samples (Student’s *t* test). Strikingly, validated pre-defined HypoM markers in cfDNA were enriched among those with larger negative methylation differences between the cancer-associated component and WBCs, while validated pre-defined HyperM markers were enriched among those with larger positive methylation differences between the cancer-associated component and WBCs (enrichment scores = 0.829 and −0.527; Kolmogorov-Smirnov test *p* value = 4.4 × 10^−16^ and 2.3 × 10^−47^, respectively; STAR Methods) (Figure 3B). This enrichment could also be observed in other components, but they were less pronounced (Figure S13). We also identified cancer-associated components in other cancer types and demonstrated the enrichment of the cfDNA-validated markers among the top differential markers based on comparisons between the cancer-associated components and WBCs (Figures 3C and S9B–S12B). These results supported the notion that cancer-specific component(s), which exhibited differential methylation profiles compared with the blood background, potentially played a dominant role in the final cancer signals detected in blood.

### Markers identified via a decomposition framework exhibit enhanced cancer signal

We have demonstrated that conventionally selected markers from tissue bulk data were more likely to be validated in blood samples when they exhibited pronounced methylation differences between cancer-associated component(s) and WBCs—an essential criterion for detectability in cfDNA. To further evaluate the utility of component-level marker selection, we conducted a head-to-head comparison between our decomposition-based approach and conventional methods, using bulk tissue data from patients with pathologically confirmed colorectal cancer (STAR Methods). Specifically, we identified the top 1,000 HyperM and 1,000 HypoM markers (including both DMCs and DMRs) using customized pipelines (Figure S14) and assessed their performance in an independent cfDNA RRBS dataset^21^ by measuring methylation differences between cancer and normal samples. This cfDNA dataset served as a proxy for blood-based cancer signal detection. Remarkably, the top markers identified by NMF decomposition exhibited significantly greater methylation differences in cfDNA than those conventionally selected using several different methods (Figure 4A). This result indicated that our component decomposition-based framework enhances the identification of more robust and distinguishable cancer markers from tissue bulk data.

**Figure 4:**
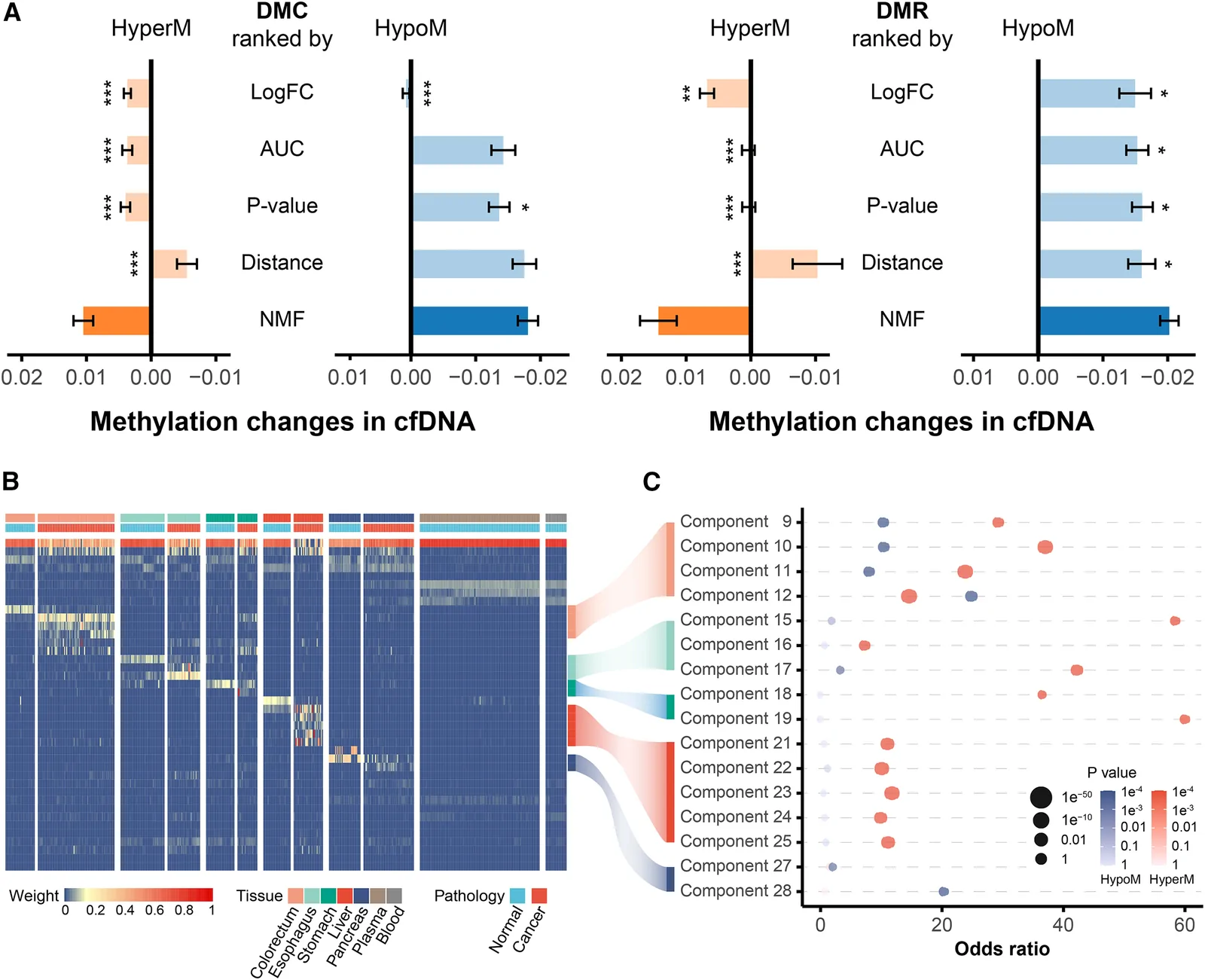
Improvement of marker identification through component decomposition (A) Comparisons between cancer markers (both the top 1,000 DMCs and top 1,000 DMRs) selected through component decomposition (NMF) and conventional approaches (distance, *p* value, AUC, log-fold-change [LogFC]) by calculating the methylation differences in cfDNA samples between cancer and normal participants. Data are represented as mean ± SEM. Significance: ∗*p* < 0.05, ∗∗*p* < 0.01, ∗∗∗*p* < 0.001 by *t* test. (B) The weight matrix (*W* matrix) of cancer and para-carcinoma samples for the five GI cancers, along with healthy plasma and blood cell samples. (C) Validation of the TOO DMCs identified through corresponding cancer type-specific components. Hypergeometric test was performed for the TOO DMCs represented by each specific component, using cfDNA-validated TOO markers from an existing panel for multi-cancer detection. The *p* values are represented by both the point size and color intensity. See also Figures S14–S16; Table S1.

Furthermore, to evaluate the improvement by the decomposition-based marker selection in cancer prediction, we constructed random forest models using the top DMC or DMR identified by the NMF-based framework and, for comparison, those selected by the conventional pipeline, based on the cfDNA RRBS dataset.^21^ Cross-validation showed that NMF-based DMC markers achieved an area under the receiver operator characteristic curve (AUC) of 0.820, significantly outperforming conventionally selected markers (AUC = 0.573–0.698; all *p* < 0.05 by DeLong test) (Figure S15A). Consistently, models using NMF-derived DMR markers yielded a higher AUC of 0.813 compared with conventional approaches (AUC = 0.651–0.690; all *p* < 0.05) (Figure S15B). Taken together, these findings from an independent external cfDNA dataset confirm that the NMF-based framework outperforms the conventional strategies assessed here in terms of marker selection for cancer prediction. However, prospective validation of an optimized assay designed in accordance with our framework is still required to definitively establish its clinical utility.

### The decomposition framework improves TOO marker selection

Building on the identification of cancer- and normal-associated component(s), we attempted to extend our framework for the selection of TOO markers, which are essential for developing an MCED approach.^1^ To extract pure components for target tissue types, including both normal and cancer cells, we applied our decomposition approach to an integrated dataset comprising bulk RRBS data from cancer and para-carcinoma samples of five GI cancers, alongside 114 healthy plasma samples and 20 healthy WBC samples as background controls. A total of 40 components were derived (Figure S16; STAR Methods). Interestingly, despite the common components detected for the blood background (e.g., components 1 and 6–8) and for normal GI tissues (e.g., components 3 and 4), we detected one component (component 2) that was prominently shared by all GI cancers, likely representing a common cancer signal. We also identified several TOO-specific components potentially indicative of different cancer types (components 9–12 for colorectal malignancy, 15–17 for esophageal, 18 and 19 for stomach, 21–25 for liver, and 27 and 28 for pancreatic) (Figure 4B). Remarkably, these TOO-specific components were well separated from the blood components, the common normal GI components, and even the common GI cancer component. Interestingly, the TOO-specific components could be associated with both normal samples (e.g., component 9) and cancer samples (e.g., components 10–12), suggesting that both normal and cancer cells provided information about the TOO localization.

We then identified TOO markers, defined as CpGs differentially methylated in one GI cancer type compared with the others, based on the methylation profiles of the TOO-specific components. Specifically, for each TOO component of a given cancer type, its methylation profile was compared pairwise with all TOO components of the other cancer types. The CpGs with the smallest methylation differences that were larger than a given cut-off (e.g., 0.1) were identified as TOO markers, with further classification as HyperM or HypoM markers according to the direction of methylation change (STAR Methods). Subsequently, the TOO markers were assessed using plasma-validated TOO markers derived from the target panel, which had been tested on thousands of cfDNA samples.^6^ Significant overlaps were observed between the two sets of TOO markers across almost all the five GI cancers (Figure 4C; Table S1). These findings support both the biological relevance of the component-derived markers and the value of the decomposition-based framework for prioritizing TOO markers. Nevertheless, the observed concordance with a previously validated plasma marker set provides only indirect evidence; direct assessment of the newly identified TOO markers’ discriminatory performance in an independent clinical cohort remains outstanding.

### Extension of the decomposition framework to transcriptomic data

To evaluate the generalizability of our framework beyond epigenetic markers, we extended the component decomposition approach to bulk transcriptomic data. RNA-seq data for five GI cancers were obtained from TCGA (The Cancer Genome Atlas) database, and for each cancer type, tumor and para-carcinoma samples were jointly decomposed using NMF based on TPM (transcripts per million) values (STAR Methods). This yielded a gene expression matrix (*H* matrix) capturing component-specific transcriptional profiles and a corresponding weight matrix (*W* matrix) reflecting the proportional contribution of each component to individual samples (Figure S17).

We then assessed whether component-based decomposition could improve RNA marker selection for blood-based cancer detection. A head-to-head comparison using TCGA bulk RNA-seq data were conducted between markers selected based on conventional methods and our decomposition approach (STAR Methods). The top 100 RNA markers from each method were evaluated in an independent cfRNA-seq dataset,^22^ by measuring the increase of the selected RNA markers between cancer and normal cfRNA samples (Figures 5A and S18). Remarkably, markers identified via NMF decomposition exhibited significantly stronger discrimination between cancer and normal cfRNA samples, indicating superior performance of the decomposition framework in cfRNA marker discovery.

**Figure 5:**
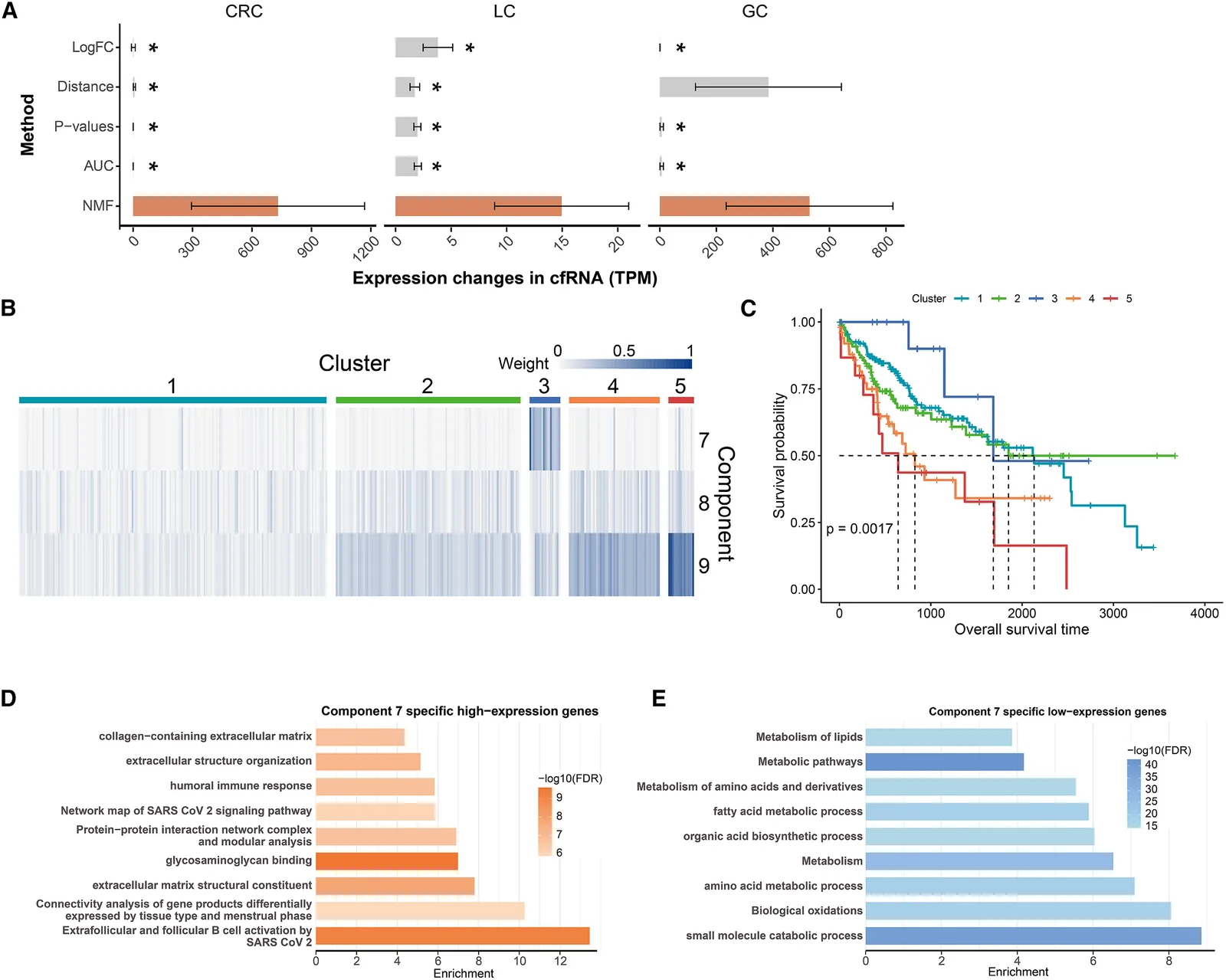
Improvement of cfRNA cancer marker identification and cancer subtype clustering by decomposition of transcriptomic data (A) Comparisons between cancer markers (both the top 100 highly expressed genes) selected through component decomposition (NMF) and conventional approaches (AUC, *p* value, distance, and log-fold-change [LogFC]) by calculating the increase of expression in cfRNA samples between cancer and normal participants. Data are represented as mean ± SEM. Significance: ∗*p* ≤ 0.05 by *t* test. The evaluation was performed for colorectal cancer (CRC), liver cancer (LC), and gastric cancer (GC). (B) Clustering of liver cancer patients based on cancer-associated components. (C) Overall survival of different clusters of liver cancer patients. (D and E) The cancer subtype (cluster 3) associated component (component 7) based differential genes. Functional enrichment was evaluated for the subtype-specific top 500 highly (D) and low (E) expressed genes. See also Figures S17–S18.

Beyond early detection, we explored whether component analysis could reveal clinically relevant patient subtypes. In liver cancer, three components (components 7–9) enriched in tumor samples were used to stratify patients into five clusters based on component weights (Figure 5B). Survival analysis revealed significant differences among the clusters (log rank *p* = 0.0017) (Figure 5C). Notably, clusters 1 and 2, with minimal contribution from tumor components, exhibited favorable survival (median overall survival [OS] time of 2,131 and 1,852 days, respectively), while clusters 4 and 5, dominated by component 9, had markedly worse outcomes (median OS time of 827 and 643 days, respectively), indicative of the malignance of component 9. Interestingly, cluster 3, characterized by a high proportion of component 7, showed an improved prognosis (median OS time of 1,685 days) against clusters 4 and 5, suggesting a potentially protective role of this component. To elucidate the underlying mechanism, we compared the transcriptomic profile of component 7 against the malignant component 9. Genes highly expressed in component 7 were enriched in immune activation pathways, including extrafollicular and follicular B cell activation and extracellular matrix remodeling (Figure 5D). Conversely, genes downregulated in component 7 were associated with lipid and amino acid metabolism and oxidative processes, suggesting attenuated tumor proliferation (Figure 5E). These results highlight the ability of transcriptomic decomposition to identify molecular subtypes with distinct immunobiological states and prognostic relevance, underscoring its potential in refining cancer classification and therapeutic stratification.

## Discussion

This study serves as a methodological framework for a broader MCED research program and plays a transitional role in advancing the next stage of MCED development. The present work is dedicated to improving the methodological foundation for marker selection, building upon high-quality data (with samples carefully balanced and processed under GCP-compliant, standardized procedures) (ClinicalTrials.gov identifier: NCT05431621).^6^ Leveraging this well-controlled dataset, we focus on extracting cancer-specific signals through data decomposition, with the aim of improving marker specificity and robustness. The methodological advances presented here directly inform and support the evolution of MCED strategies, facilitating their further optimization for population-based screening, risk-adapted surveillance, and early diagnostic evaluation in subsequent research phases.

Marker discovery is a critical step in developing effective early cancer detection panels. However, a persistent challenge lies in bridging the gap between marker discovery from tissue samples and their successful application in blood-based assays. Many tissue-derived markers ultimately fail in blood due to various factors, including false positives driven by inflammation- or injury-related components rather than malignant cells, insufficient tumor signal representation in tissue, low signal-to-noise ratio in blood, or other unknown reasons. Given the complexity and cost of cell-type-resolved marker identification, we developed a component decomposition-based framework that enables marker discovery through comparisons across relatively pure components within the tissue.

Based on the decomposition of tissue bulk data, we uncovered discrepancies between markers selected from bulk and decomposed components, helping to explain the frequent failure of tissue-derived markers in blood-based validation. We herein demonstrated that the observed methylation differences were contributed by both distinctive component methylation spectra and varying fractions of the sub-tissue components within the test tissue, and thus such differences reflected compromised signals at the tissue level, biasing the identification of true markers. The framework allowed for direct comparison between target cancer components and normal controls, while simultaneously accounting for the methylation background of blood (e.g., WBCs), ultimately yielding more discriminative and blood-relevant markers.

We further extended this framework to identify TOO markers by leveraging well-separated, cancer-type-specific components that diverged not only from normal tissue and blood-derived components but also from common GI cancer components. These observations suggest that TOO information may originate from both malignant and non-malignant components within the tissue, offering a refined approach to marker discovery for MCED efforts.

Moreover, we demonstrated that the framework was applicable to other biomarkers, by the decomposition of transcriptomic data, which enabled the identification of cfRNA markers with superior performance compared to conventional selection methods. Beyond marker discovery, our analysis highlighted the potential of data decomposition not only in early detection but also in prognostic stratification and therapeutic guidance.

Our framework can be further extended to include benign lesions (e.g., polyps, cirrhosis), allowing for the identification of markers that distinguish cancer from inflammation or pre-malignancy. It can also support differential diagnosis and subtype discovery through decomposition of mixed neoplasia or cancer subtypes. Moreover, the approach can be adapted for multi-omics integration, enabling joint decomposition across DNA methylation, RNA, and protein data to capture tumor evolution at high resolution.

In conclusion, we believe that the component decomposition-based framework presented here offers a powerful strategy for marker discovery in tissue bulk data, with the potential to substantially improve the performance of liquid biopsies assays for early cancer detection.

### Limitations of the study

Several limitations may be pointed out for the present study. First, although the decomposition-based marker-selection framework yielded enhanced signal strength and prediction accuracy for cancer detection when evaluated against an external public cfDNA dataset, these optimized markers have not yet undergone experimental or prospective validation using a dedicated targeted methylation panel in a large, independent clinical cohort. Similarly, although the component-derived TOO markers correlated well with established plasma TOO markers, this concordance does not eliminate the necessity for direct empirical testing. The discriminatory capacity of these newly identified TOO markers has not been assessed in a separate clinical population. Collectively, these gaps underscore the need for rigorous experimental and prospective validation to determine whether the observed benefits are reproducible in real-world clinical specimens and to gauge the translational potential of the framework. Second, we relied solely on NMF for decomposition. While alternative methods may also be suitable, we selected NMF for its interpretability, scalability, and independence from reference datasets.^19^^,^^23^ Third, the components were not experimentally matched to specific cell types. However, our framework does not require a strict one-to-one correspondence with canonical cell identities. Instead, components can be viewed as “sub-tissues” capturing shared epigenetic or transcriptional features. Fourth, although validated in five GI cancer types, the scalability of the framework to a broader MCED setting warrants further investigation. Fifth, although this study was based on high-quality data and conducted under GCP-compliant procedures, residual pre-analytical variability may still potentially exist among samples (e.g., age, sex, sample collection, library preparation, and sequencing lanes), and NMF may in principle capture components related to such pre-analytical or confounding factors, which should be carefully interpreted in future applications. Nevertheless, we believe that the structured nature of the decomposition greatly reduces the complexity associated with multi-way comparisons inherent to conventional methods for MCED development.

## Resource availability

### Lead contact

Requests for further information and resources should be directed to and will be fulfilled by the lead contact, Xin-Rong Yang (yang.xinrong@zs-hospital.sh.cn).

### Materials availability

This study did not generate new unique reagents.

### Data and code availability

• Targeted methylation sequencing data for the cfDNA from cancer and health participants were derived from a previous study,^6^ which had been deposited at BioProject database of China National Center for Bioinformation under the Project ID PRJCA021793 (https://ngdc.cncb.ac.cn/bioproject/).
• RRBS data for five GI cancers, including cancer samples and para-carcinoma samples, healthy plasma samples, and blood cell samples analyzed in this paper were referenced from the previous study.^6^ Further details can be made available through the lead contact upon reasonable request.
• This paper analyzes existing, publicly available processed WGBS data from sorted normal tissue cells, accessible at GEO: GSE186458.
• This paper analyzes existing, publicly available processed methylation array data from cancer cell lines, accessible at GEO: GSE68379.
• This paper analyzes existing, publicly available RRBS data for the cfDNA from plasma samples collected from patients with colorectal cancer and healthy donors, accessible at the CFDA (Cell-Free Epigenome Atlas) database.
• This paper analyzed existing, publicly available transcriptomic data for the five GI cancers, including cancer samples and para-carcinoma samples, as well as the survival data of the corresponding patients, accessible at TCGA database.
• This paper analyzed existing, publicly available RNA-seq data for the cfRNA from plasma samples collected from colorectal cancer, gastric cancer, liver cancer, and healthy donors, accessible at the previous study.^22^
• The original code is deposited in GitHub at https://github.com/faculty/MarkerDecompose, which is publicly available as of the date of publication.
• Any additional information required to reanalyze the data reported in this paper is available from the lead contact upon request.

## Acknowledgments

This work was jointly supported by the 10.13039/501100012166National Key Research and Development Program of China (2024ZD0525400, 2024ZD0525405, and 2024ZD0525406), the National Basic Science Center Project of China (82488101), the 10.13039/501100001809National Natural Science Foundation of China (82341027 and 82072715), the 10.13039/501100018538National Ten Thousand Talent Program of China, the Yangzhou Lvyangjinfeng Talent Program (YZLYJFJH2023CX020 and YZLYJFJH2024CX013), the Beijing Municipal Natural Science Foundation of China (No.7232172), the Shanghai Municipal Science and Technology Major Project, the Eastern Talent Program (Leading project), the Projects from the 10.13039/501100003399Shanghai Science and Technology Commission (21140900300, 22S31901800, and 25JS2850400), the project from 10.13039/501100008750Shanghai Hospital Development Center (SHDC2023CRD025), the Project of Shanghai Municipal Health Commission (2022LJ005), and the Projects from the Science Foundation of Zhongshan Hospital, 10.13039/501100003347Fudan University (2021ZSCX28, 2020ZSLC31, and ZP2023-017).

## Author contributions

W.C. and R.-Q.F. applied the methodology and performed the primary data analysis. Y.-H.A., Z.-X.S., and H.-X.S. conceived and designed the study, and performed the interpretation. W.-J.Z., F.W., H.C., W.L., M.-J.X., and C.-C.M. curated the data and contributed to data analysis. Z.-X.S., R.L., and X.-R.Y. managed and supervised the project. W.C., Y.-H.A., R.-Q.F., H.-X.S., Z.-X.S. R.L., and X.-R.Y. wrote the manuscript. All authors contributed to editing and revising the manuscript.

## Declaration of interests

R.L. reports stock ownership in Singlera Genomics and is an employee of Singlera Genomics. Z.-X.S., R.-Q.F., H.C., W.L., M.-J.X., and C.-C.M. are employees of Singlera Genomics. All other authors declare no competing interests.

## STAR★Methods

### Key resources table

**Table:** 

REAGENT or RESOURCE	SOURCE	IDENTIFIER
**Deposited data**		

RRBS data for GI cancers and para-carcinoma tissues	Huang et al.^6^	https://doi.org/10.1186/s12943-025-02367-x
Healthy plasma RRBS data	Huang et al.^6^	https://doi.org/10.1186/s12943-025-02367-x
Healthy blood cell RRBS data	Huang et al.^6^	https://doi.org/10.1186/s12943-025-02367-x
cfDNA targeted methylation sequencing data	Huang et al.^6^	https://doi.org/10.1186/s12943-025-02367-x
cfDNA RRBS data	CFDA^21^	https://doi.org/10.1093/nar/gkz715
WGBS data from sorted normal tissue cells	Loyfer et al.^14^	GEO: GSE186458
Methylation array data from cancer cell lines	Iorio et al.^24^	GEO: GSE68379
Tissue bulk transcriptomic data	TCGA	https://gdc.xenahubs.net
Survival information	TCGA	https://gdc.xenahubs.net
cfRNA data	Wang et al.^22^	https://doi.org/10.1038/s41467-023-44461-y
Code for the decomposition of RRBS data	This paper	https://github.com/faculty/MarkerDecompose

**Software and algorithms**		

WebGestalt	Elizarraras et al.^25^	https://www.webgestalt.org/
Python	Python Software Foundation	https://www.python.org
scipy.stats module of Python	SciPy Project	https://scipy.org/
scikit-learn library of Python	Scikit-learn	https://scikit-learn.org/

### Experimental model and study participant details

#### Datasets

The present study was conducted exclusively through computational re-analysis of previously generated datasets. No new human participants were recruited, and no experiments involving animals or cell lines were performed. The datasets analyzed in this study were obtained from previously published studies and publicly accessible repositories, with the corresponding sources and accession information provided in the key resources table.

The bulk RRBS datasets included 199 gastrointestinal (GI) cancer samples, comprising 73 colorectal, 31 esophageal, 19 stomach, 28 liver, and 48 pancreatic cancer samples, together with 153 para-carcinoma tissue samples from 28 colorectal, 42 esophageal, 27 stomach, 26 liver, and 30 pancreatic cancer patients. The datasets also included 114 healthy plasma samples and 20 healthy whole-blood-cell samples. The plasma cfDNA dataset included 845 cancer patients, comprising 200 colorectal, 150 esophageal, 159 stomach, 293 liver, and 43 pancreatic cancer patients, as well as 832 healthy participants.

Because the present study involved only the re-analysis of existing de-identified datasets, the potential influence of sex and gender was not independently assessed beyond the information and study designs of the original studies. Detailed demographic characteristics of the study participants, including age, sex and/or gender, cancer stage, race, and ethnicity, where reported, are available in the corresponding original publications listed in the key resources table. No additional participant recruitment, sample collection, or experimental procedures were conducted in the present study, and therefore no additional institutional ethical approval was required for this re-analysis.

### Method details

#### Non-negative matrix factorization (NMF) of bulk RRBS data

The bulk RRBS data was subjected to NMF to decompose it into components based on the model:

(Equation 1) R=HW+ε

using the detailed formula as follows:

(Equation 2) $$\left(\begin{array}{cccc} r_{11} & r_{12} & \cdots & r_{1N}\\ r_{21} & r_{22} & \cdots & r_{2N}\\ \vdots & \vdots & \ddots & \vdots \\ r_{M1} & r_{M2} & \cdots & r_{MN}\\ e_{1} & e_{2} & \cdots & e_{N} \end{array}\right)=\left(\begin{array}{ccc} h_{11} & \cdots & h_{1S}\\ h_{21} & \cdots & h_{2S}\\ \vdots & \ddots & \vdots \\ h_{M1} & \cdots & h_{MS}\\ e_{1}^{\prime } & \cdots & e_{S}^{\prime } \end{array}\right)\left(\begin{array}{cccc} w_{11} & w_{12} & \ldots & w_{1N}\\ \vdots & \vdots & \ddots & \vdots \\ w_{S1} & w_{S2} & \cdots & w_{SN} \end{array}\right)+\varepsilon$$

The matrix *R* is the bulk RRBS data of *N* samples (column) with *M* CpGs (row), in which the *r*_*ij*_ (1 ≤ *i* ≤ *M*, 1 ≤ *j* ≤ *N*) is the observed methylation value of CpG *i* in Sample *j*. The matrix *H* is the methylation profile matrix of *S* components (column) with *M* CpGs (row), in which the *h*_*ik*_ (1 ≤ *i* ≤ *M*, 1 ≤ *k* ≤ *S*) represents the methylation level of CpG *i* in Component *k*. The matrix *W* is the weight matrix of the *S* components (row) in *N* samples (column), in which the *w*_*kj*_ (1 ≤ *k* ≤ *S*, 1 ≤ *j* ≤ *N*) indicates the weight, which can be treated as the fraction, of Component *k* in Sample *j*. The matrix *ε* is the residue of the decomposition and could be ignored for simplicity. Therefore, the methylation value of CpG *i* in Sample *j* at the bulk level, namely *r*_*ij*_, can be calculated as the sum of the methylation levels of CpG *i* across all components (*h*_*ik*_, 1 ≤ *k* ≤ *S*) weighted by the fraction of the corresponding component(s) in Sample *j* (*w*_*kj*_, 1 ≤ *k* ≤ *S*) as follows:

(Equation 3) $$r_{ij}=\sum_{k=1}^{S}h_{ik}w_{kj}+\varepsilon_{ij}$$

Please note that an artificial CpG was added at the bottom row of matrix *R* as an internal reference, where all *e*_*j*_ (1 ≤ *j* ≤ *N*) were set to 1, indicating full methylation across all samples. Consequently, the methylation profile of the decomposed components can be scaled to 0–1, by maintaining the methylation value of the artificial CpG as 1 across all components ($e_{k}^{\prime }=1$, 1 ≤ *k* ≤ *S*). Meanwhile, the sum of weights across samples of each component was normalized to 1 (*i*.*e*., 100%) because

(Equation 4) $$e_{j}\approx \sum_{k=1}^{S}e_{k}^{\prime }w_{kj}=\sum_{k=1}^{S}\left(1\times w_{kj}\right)=1\times \left(\sum_{k=1}^{S}w_{kj}\right)$$

Therefore, when *e*_*j*_ = 1 (1 ≤ *j* ≤ *N*), the sum of weights across all the components (1 ≤ *k* ≤ *S*) in Sample *j* $\sum_{k=1}^{S}w_{kj}\approx 1$ (1 ≤ *j* ≤ *N*).

The NMF was performed by implementing the NMF function from the sklearn.decomposition module in the scikit-learn library of Python.

#### Quantification of the contribution to bulk data by each component

Each component contributing to the observed methylation data at the bulk level could be quantified through matrix calculations:

(Equation 5) $$\left(\begin{array}{c} h_{1k}\\ h_{2k}\\ \vdots \\ h_{Mk} \end{array}\right)\left(\begin{array}{cccc} w_{k1} & w_{k2} & \cdots & w_{kN} \end{array}\right)=\left(\begin{array}{cccc} h_{1k}w_{k1} & h_{1k}w_{k2} & \cdots & h_{1k}w_{kN}\\ h_{2k}w_{k1} & h_{2k}w_{k2} & \cdots & h_{2k}w_{kN}\\ \vdots & \vdots & \ddots & \vdots \\ h_{Mk}w_{k1} & h_{Mk}w_{k2} & \cdots & h_{Mk}w_{kN} \end{array}\right)$$

for Component *k* (1 ≤ *k* ≤ *S*), in which the vector *h*_*ik*_ (1 ≤ *i* ≤ *M*) is the methylation spectrum across all the CpGs in Component *k* (the *k*-th column in matrix *H*), and the vector *w*_*kj*_ (1 ≤ *j* ≤ *N*) is the weight of Component *k* across all the samples (the *k*-th row in matrix *W*). In the product matrix, every value *h*_*ik*_*w*_*kj*_ (1 ≤ *i* ≤ *M*, 1 ≤ *j* ≤ *N*) represents the methylation of CpG *i* in Sample *j* contributed by Component *k*.

#### Decomposition using cancer and control samples

The RRBS data for both cancer and para-carcinoma samples were merged for decomposition for each cancer type. The CpGs with at least 10× reads in ≥70% samples and with an AUC (area under receiver operating characteristic [ROC] curve) of ≤35% or ≥65% across the samples were selected for decomposition. Based on the pre-selected CpGs, the raw methylation values of cancer and para-carcinoma samples were combined into a single matrix, on which NMF was implemented to generate around 8–12 components. The optimal number of components was determined via a two-step decomposition: a coarse decomposition with around 5–6 components followed by a fine decomposition with about 8–10 components for undetermined components, or vice versa. The second round of decomposition was implemented on the recovered matrix, which was the product of the *H* matrix and *W* matrix of the undetermined components, with the addition of the residuals.

To determine the condition (*i*.*e*., cancer or normal)-associated components, the fractions of each component were compared between cancer and normal samples using a two-sided Student’s *t* test.

#### Tissue or cell type identification for components

Processed WGBS data from sorted normal tissue cells (GEO accession number GSE186458)^14^ and processed methylation array data from cancer cell lines (GEO accession number GSE68379)^24^ were used to identify the tissue/cell type corresponding to each component. For each component, Pearson correlation coefficients (PCCs) were calculated between its methylation profile and those of various reference tissue/cell types. The reference tissue/cell type yielding a relatively high PCC was considered the type associated with the component.

#### Conventional identification of cancer markers from tissue bulk data

A sliding window of 200 bp, encompassing at least one CpG, was used to detect cancer markers from tissue bulk data. The methylation level of each window was measured using the average methylation fraction (AMF), calculated as the ratio of methylated reads to total reads within the window region. Then, differential windows were identified via a two-sided Mann-Whitney U-test, fold-change, or the distance of median AMFs between cancer and normal samples. Overlapping significantly differential windows showing consistent methylation alterations were merged as cancer hyper-methylated (HyperM) markers and cancer hypo-methylated (HypoM) markers, as appropriate. In order to validate the detected markers in blood samples, we further overlapped these window-based markers with an existing panel for multi-cancer early detection, which has been tested with over a thousand blood samples,^6^ to generate a subset of high quality markers.

#### Enrichment analysis of cfDNA validations for the component-based marker selection

The subset of cancer markers conventionally selected from tissue bulk data was further validated in blood samples^6^ by assessing the significance of the cfDNA methylation differences between normal and cancer samples. These markers were ranked based on the average methylation differences, calculated between each component decomposed from the RRBS data and the 20 healthy blood samples, separately for HyperM and HypoM markers. An enrichment score and the Kolmogorov-Smirnov test were used to assess whether the cfDNA validated markers were enriched at the top of the ranked list, indicating that the markers with larger differences at the component level were more likely to be validated by cfDNA.

#### Head-to-head comparison of cancer methylation markers selected through the decomposition-based framework versus conventional approaches

The cancer-specific component was first determined from the decomposed data for CRC following the above method, and the markers were compared to those in the healthy blood samples in terms of the methylation levels across all the CpGs. The top 1,000 differential sites (*i*.*e*., DMCs) were then identified as cancer-component markers for CRC. Meanwhile, we also conventionally selected the top 1,000 DMCs based on the AUC, methylation difference, fold-change and Mann-Whitney U-test between cancer and normal tissue samples. To evaluate these markers, we calculated their methylation differences between CRC and healthy samples using independent RRBS cfDNA data from the CFDA,^21^ and compared the results between our decomposition-based framework and each conventional approach. In addition to the DMCs, we also identified differential methylation regions (DMRs) using a sliding window of 200 bp, and evaluated them using the same datasets as the DMCs. To amplify the methylation signal in cfDNA, RRBS cfDNA data were pooled to calculate the methylation value for each cancer marker.

#### Decomposition of multi-cancer data and identification of TOO markers

The RRBS data for cancer and para-carcinoma samples from all five GI cancers, along with RRBS data from healthy plasma and healthy WBCs, were combined for decomposition. CpGs with at least 10× reads in ≥50% samples from each of the five cancer groups and each of the five para-carcinoma groups that showed significant differences among the five cancer groups or among the five para-carcinoma groups (*p*-value <0.05 by ANOVA), were selected for decomposition. The raw matrix was then subjected to NMF, and was decomposed into 40 components. For each cancer type, any component with a sample fraction significantly higher than that of the other cancer types was considered to be a specific component for the given cancer.

TOO markers were identified for each cancer type using each of its specific component(s). Specifically, the methylation spectrum for each TOO component was compared with all TOO components for the other cancer types. CpGs with maximal differences ≤ −0.1 or minimal differences ≥0.1 were considered to be TOO HypoM and HyperM markers, respectively.

To validate the TOO markers, we utilized an existing panel for multi-cancer detection to examine the enrichment of the decomposition-based TOO markers among the pre-defined TOO markers for corresponding cancers. A one-sided hyper-geometric test was performed separately for HyperM and HypoM markers, based on (i) all >36,000 CpGs in the panel (the full set), (ii) CpGs in target markers for each cancer type, (iii) TOO DMCs identified based on component decomposition covered by the panel, and (iv) the overlap of (ii) and (iii). The hyper-geometric test helped to determine whether the decomposition-based TOO markers were significantly enriched in the pre-defined target markers for each cancer type, thus validating their relevance and potential utility in multi-cancer detection.

#### NMF of bulk transcriptomic data

The NMF of bulk transcriptomic data was performed using a similar model as that of RRBS data. Briefly, the normalized transcripts per million (TPM) values of genes were used as input, with an artificial reference gene added—assigned a constant TPM equal to the average expression level of the ACTB (β-actin) gene across all samples. The resulting data matrix was then decomposed into *H* and *W* matrices.

#### Head-to-head comparison of cancer RNA markers selected through the decomposition-based framework versus conventional approaches

The decomposition for bulk transcriptomic data of cancer and para-carcinoma was conducted, yielding nine components. The maximum values across the nine components were used to rank the genes, of which the top 100 were identified as cancer markers. Meanwhile, conventional marker selection was performed by identifying the top 100 highly expressed genes based on group-wise comparisons between cancer and normal tissues, using metrics such as AUC (Area Under Receiver Operator Characteristic Curve), expression difference, fold-change, and the *p*-value from the Mann–Whitney U test. To evaluate these RNA markers, we calculated their expression differences between cancer and healthy samples using an independent cfRNA-seq dataset,^22^ and compared the results from our decomposition-based framework with those from each conventional approach (Figure S18).

#### Subtype identification and functional analysis based on decomposed transcriptomic data

After the decomposition of the liver cancer transcriptomic data, cancer enriched components were determined by comparing the fractions between cancer and para-carcinoma samples using a two-sided *t* test with adjusted *p*-value <0.05. Then, patients were clustered into five groups, based on which, a survival analysis was performed by using Kaplan-Meier method. Clusters exhibiting distinctive fraction patterns and unique survival outcomes were determined as potential cancer subtypes, with the corresponding enriched components defined as subtype-specific. Finally, the transcription profile of each subtype specific component was compared against other cancer-enriched components as background. The top 500 upregulated and 500 downregulated genes were subjected to functional enrichment analysis using WebGestalt (https://www.webgestalt.org/) online tool,^25^ yielding subtype-specific biological functions.

### Quantification and statistical analysis

#### Statistical analyses

Hypothesis tests were conducted using the scipy.stats module (scipy Version 1.5.4) in Python (Version 3.6.9), with specified methods indicated for the different analyses. Comparisons of continuous variables between two groups were performed using t-test or Mann–Whitney U test, as appropriate. Comparisons of continuous vaiables among three or more groups were performed using one-way ANOVA. The enrichment of a subset within the ranked list was assessed using Kolmogorov–Smirnov test. The Benjamini-Hochberg (BH) method was used for multiple test correction, particularly for determining bulk tissue markers, with an adjusted p-value cut-off of 0.05, unless otherwise specified. Statistical significance: ∗p < 0.05, ∗∗p < 0.01, ∗∗∗p < 0.001, ∗∗∗∗p < 0.0001. Differential signals were evaluated comprehensively based on p-value, area under the receiver operator characteristic curve (AUC), distance, and log-fold change.
