# Supplementary material for: Decomposition-driven biomarker identification enhances non-invasive early cancer detection
Source: iScience. 2026 Sep 24;29(10):117621. doi: 10.1016/j.isci.2026.117621 (PMC13634983; doi:10.1016/j.isci.2026.117621)
Supplement: Supplementary file 1 [file mmc1.pdf]

iScience, Volume *29*

Supplemental information

Decomposition-driven biomarker identification enhances non-invasive early cancer detection

Weizhong Chen, Ying-Hong An, Rui-Qing Fu, Hai-Xiang Sun, Wen-Jing Zheng, Fang Wang, Hua Chen, Wei Li, Min-Jie Xu, Cheng-Cheng Ma, Zhi-Xi Su, Rui Liu, and Xin-Rong Yang

Supplemental Figures

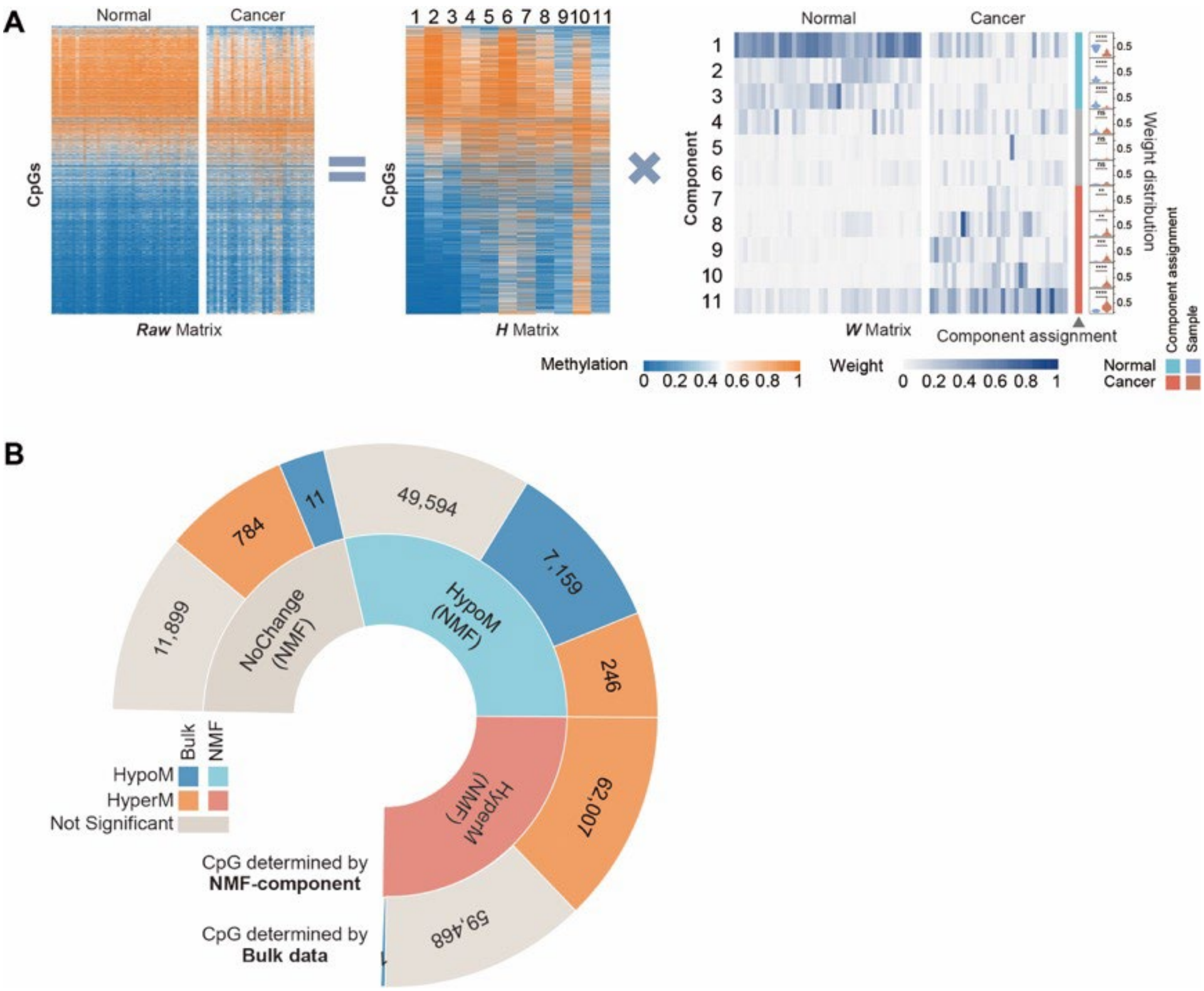

Figure S1. Decomposition of bulk DNA methylation data for esophageal cancer, Related to Figure 1.

(A) The decomposition of esophageal cancer and para-cancer RRBS data resulted in eleven components, represented as the product of the *H* matrix and the *W* matrix. Each component was assigned to either a normal or cancer condition, according to the distribution of component weights across the samples (by t-test). **(B)** Comparison of CpG alteration patterns between esophageal cancer and normal esophagus before and after decomposition. CpGs were defined as hyper-methylated (HyperM) or hypo-methylated (HypoM) markers in bulk data if they were significantly hyper-methylated or hypo-methylated in cancer samples ($p < 0.05$; U-test followed by Benjamini-Hochberg correction), and were defined as HyperM or HypoM markers if their $\log_2$(fold-change) was > 0.1 or < -0.1 between the average methylation of cancer-associated components and that of normal-associated components. The numbers (labelled in the plot) were $\log_{10}$-scaled for display. Significance: * $p < 0.05$, ** $p < 0.01$, *** $p < 0.001$, **** $p < 0.0001$.

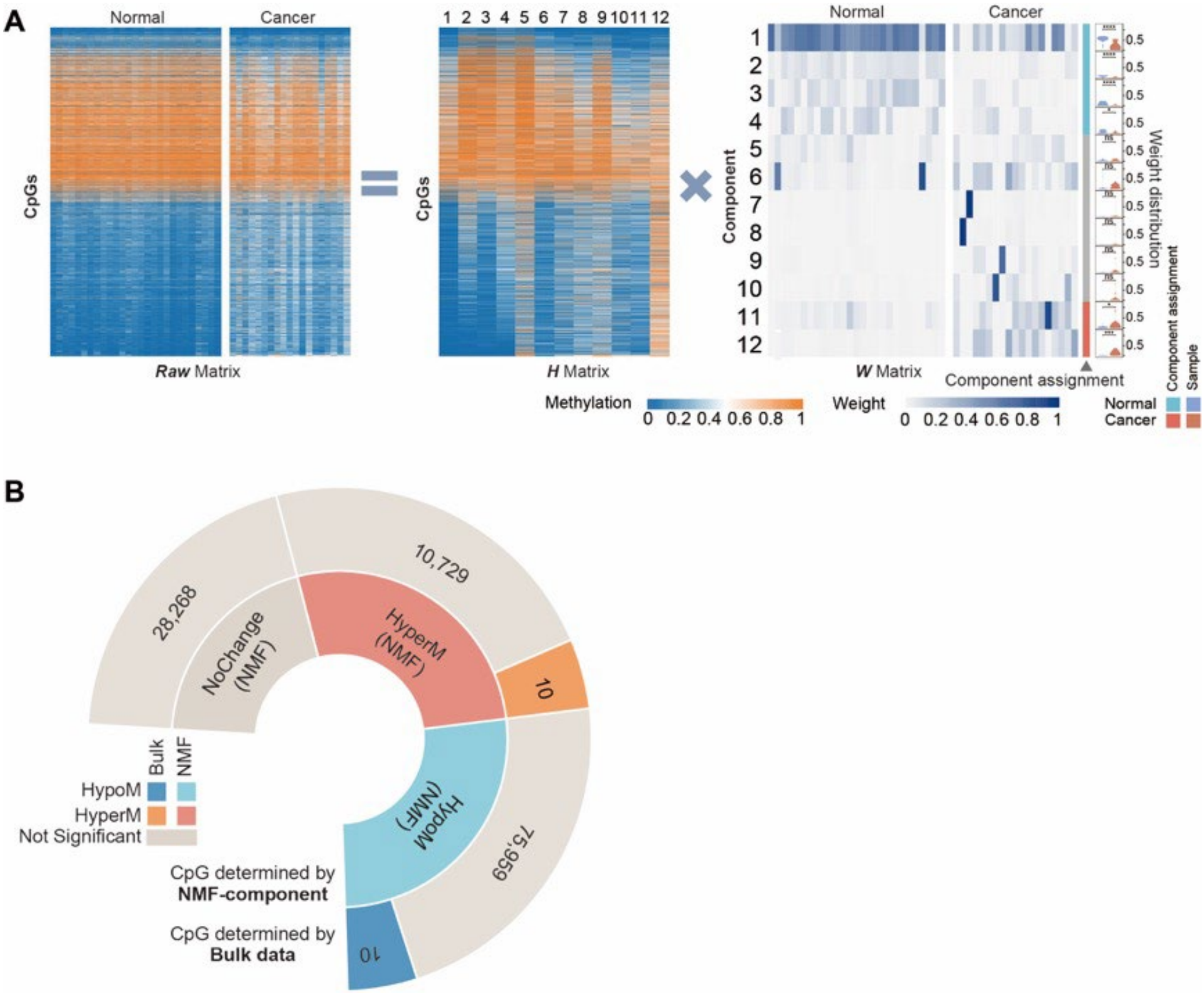

Figure S2. Decomposition of bulk DNA methylation data for gastric cancer, Related to Figure 1.

The same descriptions as in Figure S1, but for gastric cancer.

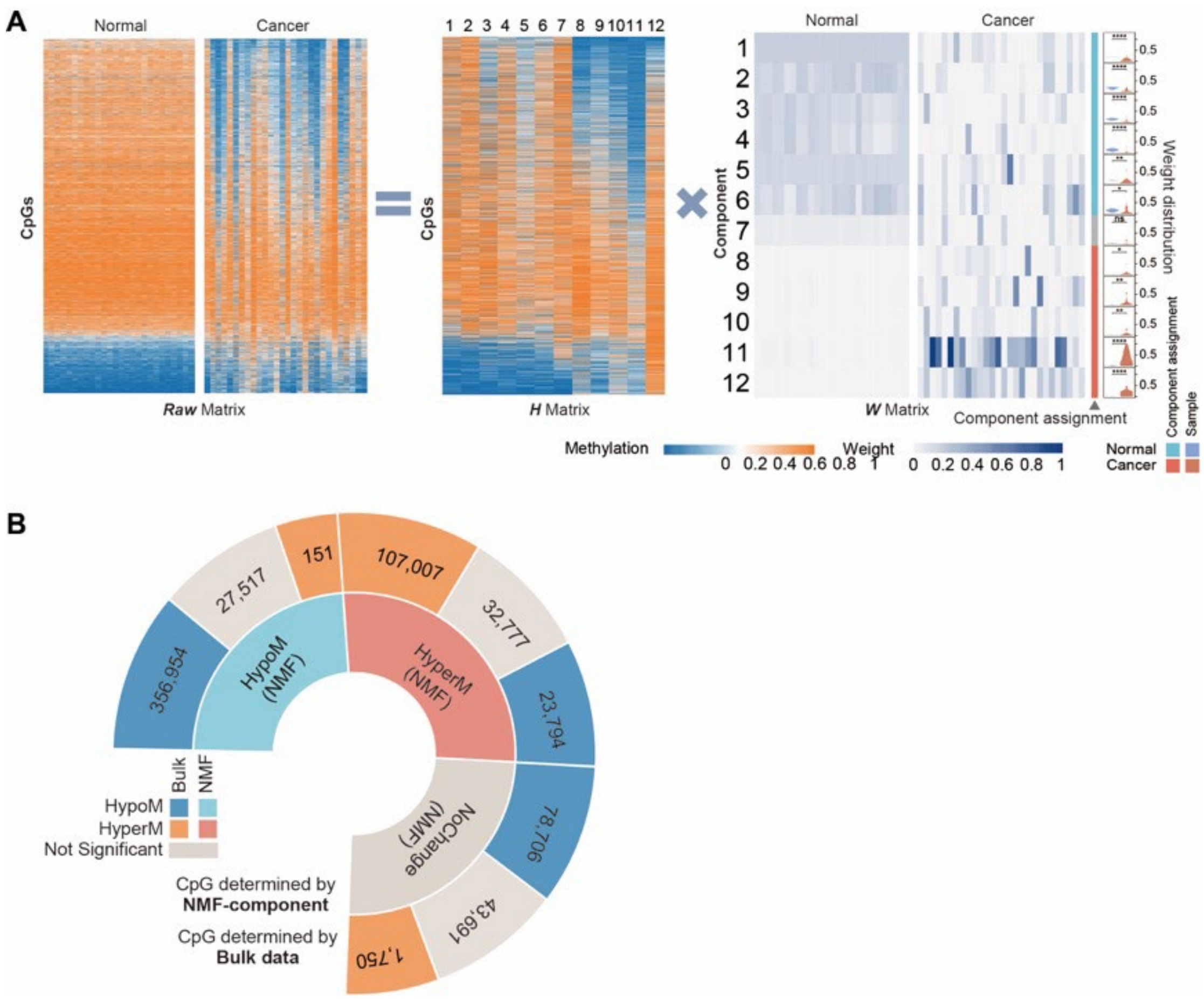

Figure S3. Decomposition of bulk DNA methylation data for liver cancer, Related to Figure 1.

The same descriptions as in Figure S1, but for liver cancer.

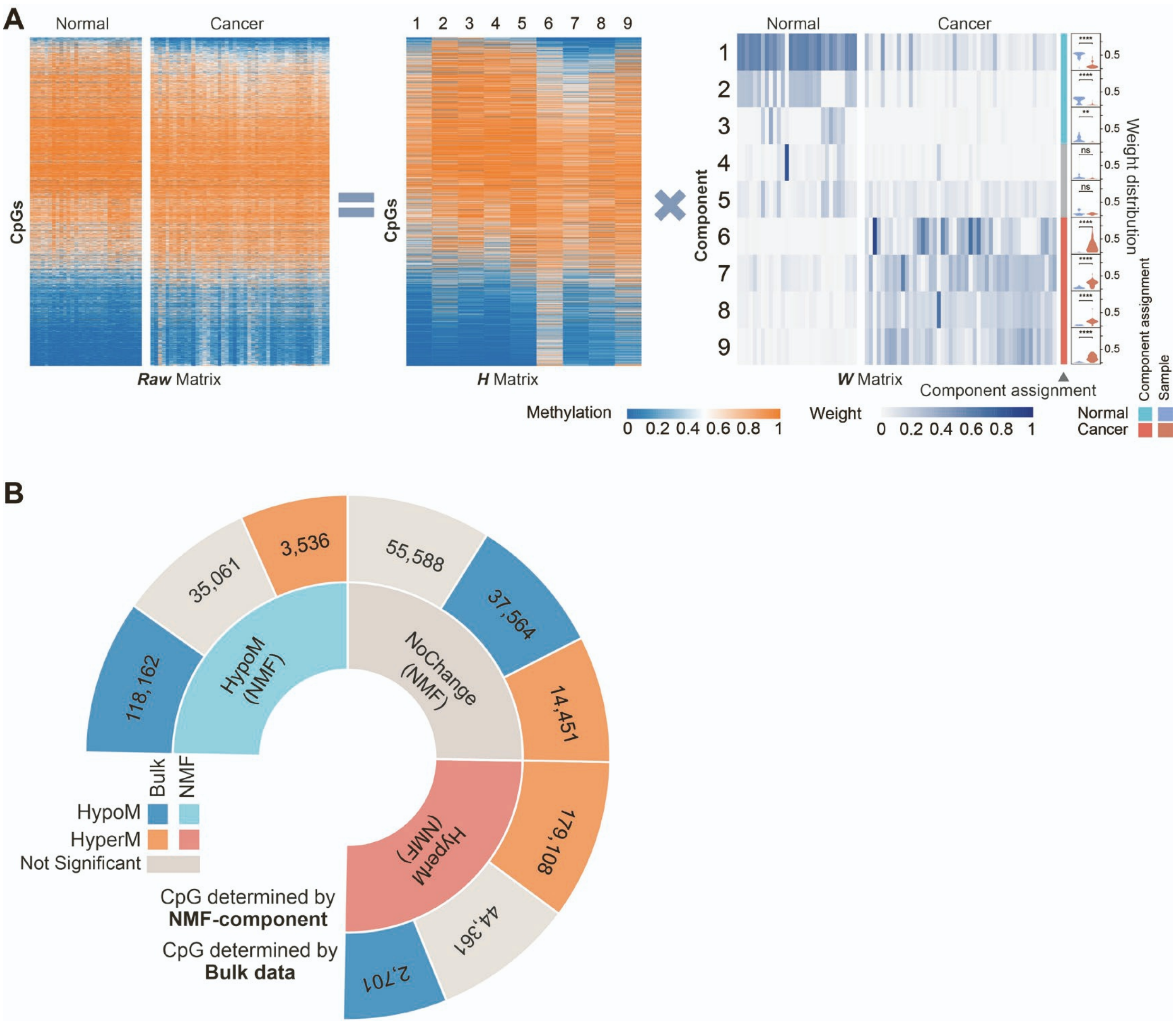

Figure S4. Decomposition of bulk DNA methylation data for pancreatic cancer, Related to Figure 1.

The same descriptions as in Figure S1, but for pancreatic cancer.

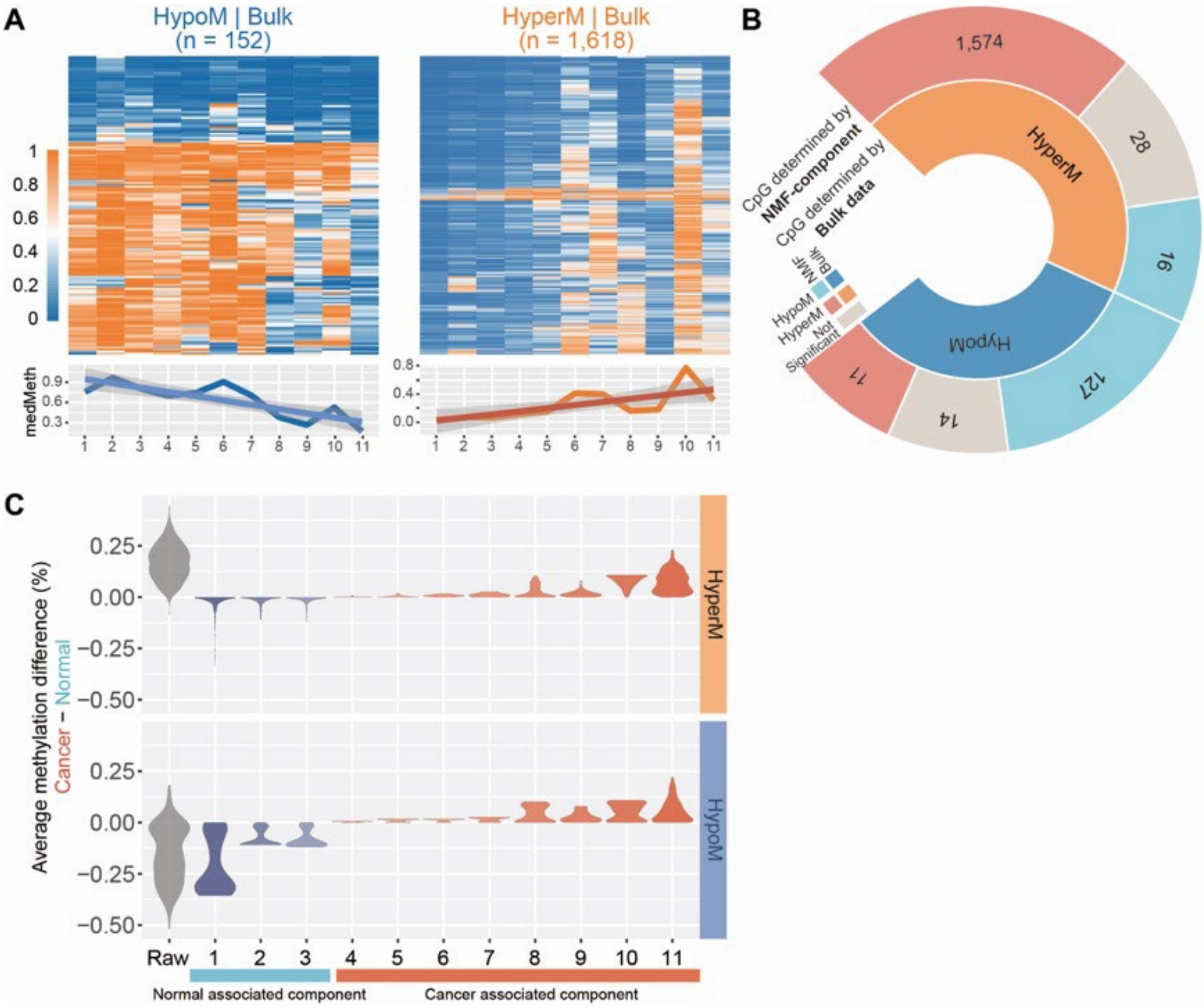

Figure S5. Component contributions to esophageal tissue markers, Related to Figure 2. (A) Methylation profiles of tissue cancer markers identified from the RRBS esophageal data across components, with median methylation values shown at the bottom. **(B)** Examination of methylation alteration patterns of tissue cancer markers at the component level. **(C)** The distribution of average methylation differences between cancer and normal samples for HyperM and HypoM tissue markers, respectively, calculated using raw RRBS data, and each of the component spectra. The component contribution was calculated as the inner product of the methylation spectrum of a certain component and its weights across samples, and average differences were calculated between cancer and normal samples.

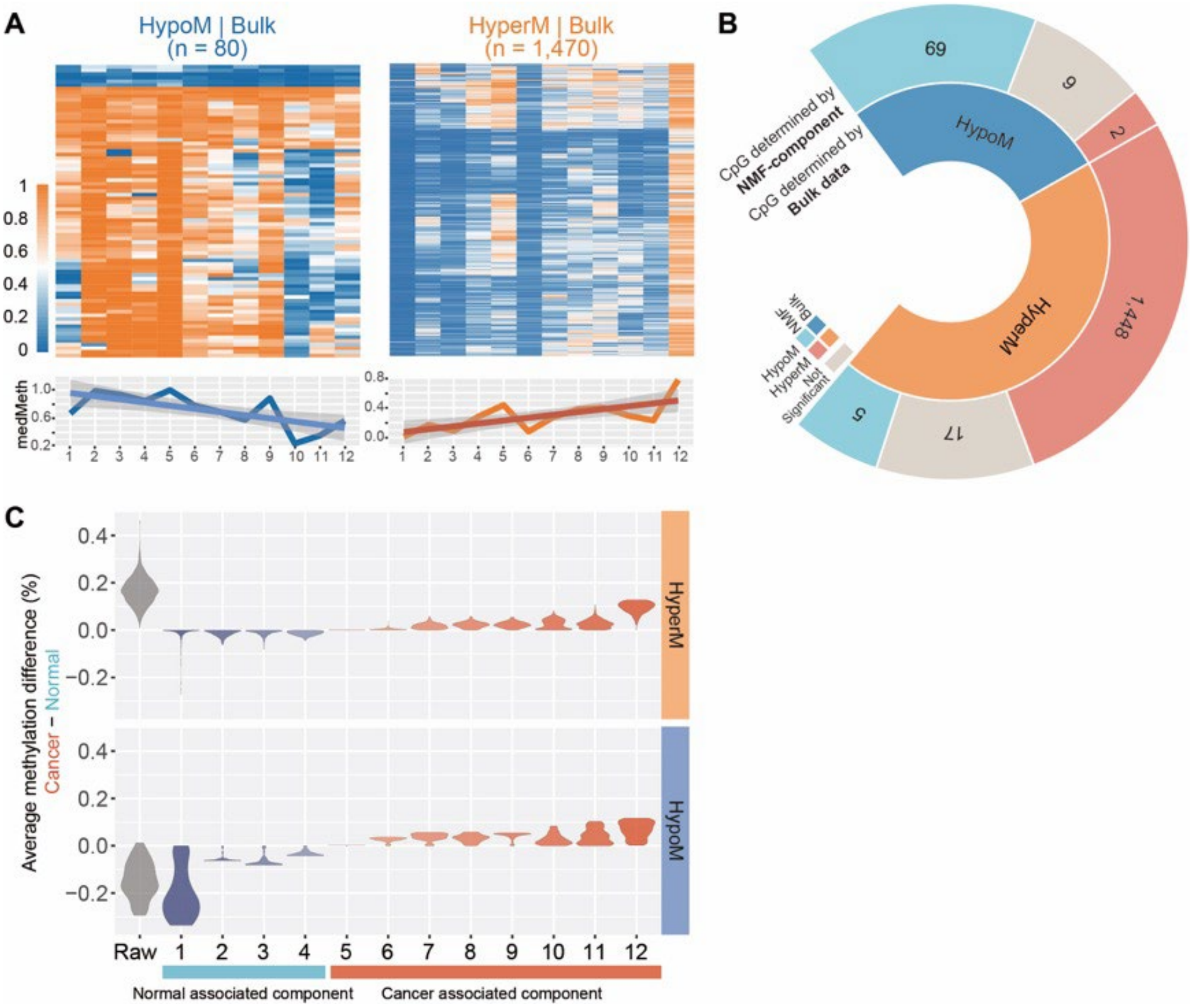

Figure S6. Component contributions to gastric tissue markers, Related to Figure 2.
The same descriptions as in Figure S5, but for gastric cancer.

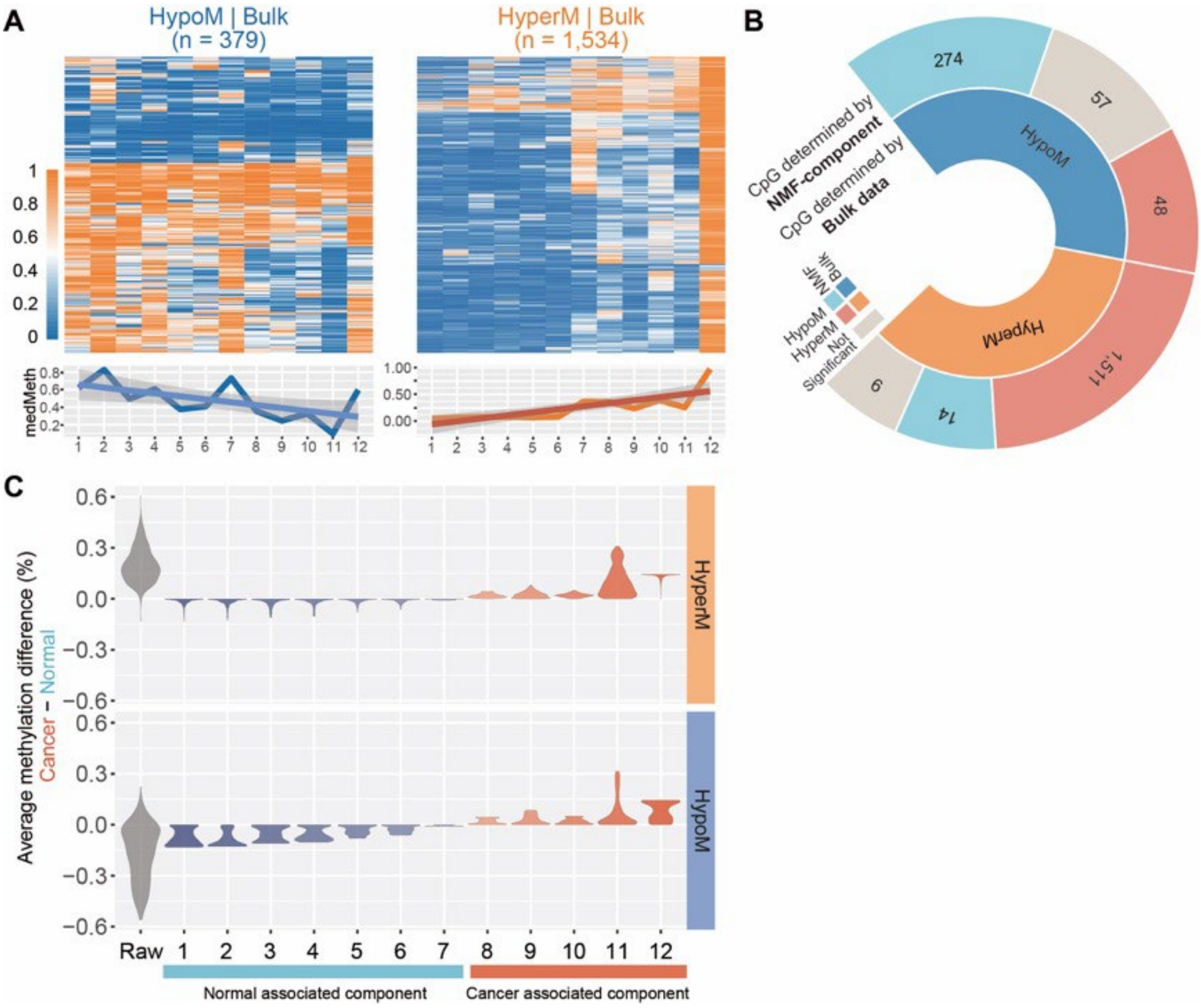

Figure S7. Component contributions to liver tissue markers, Related to Figure 2.

The same descriptions as in Figure S5, but for liver cancer.

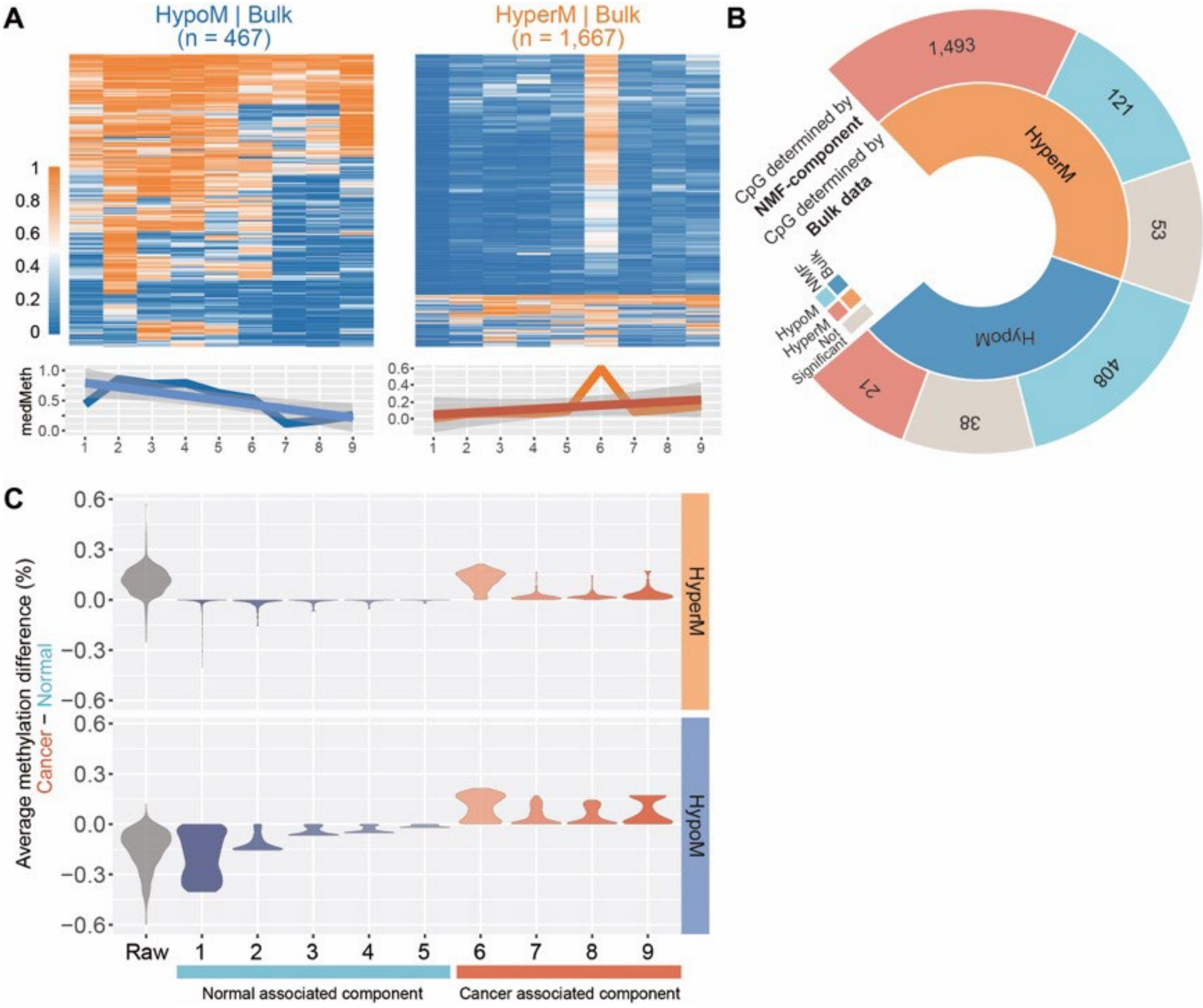

Figure S8. Component contributions to pancreatic tissue markers, Related to Figure 2.
The same descriptions as in Figure S5, but for pancreatic cancer.

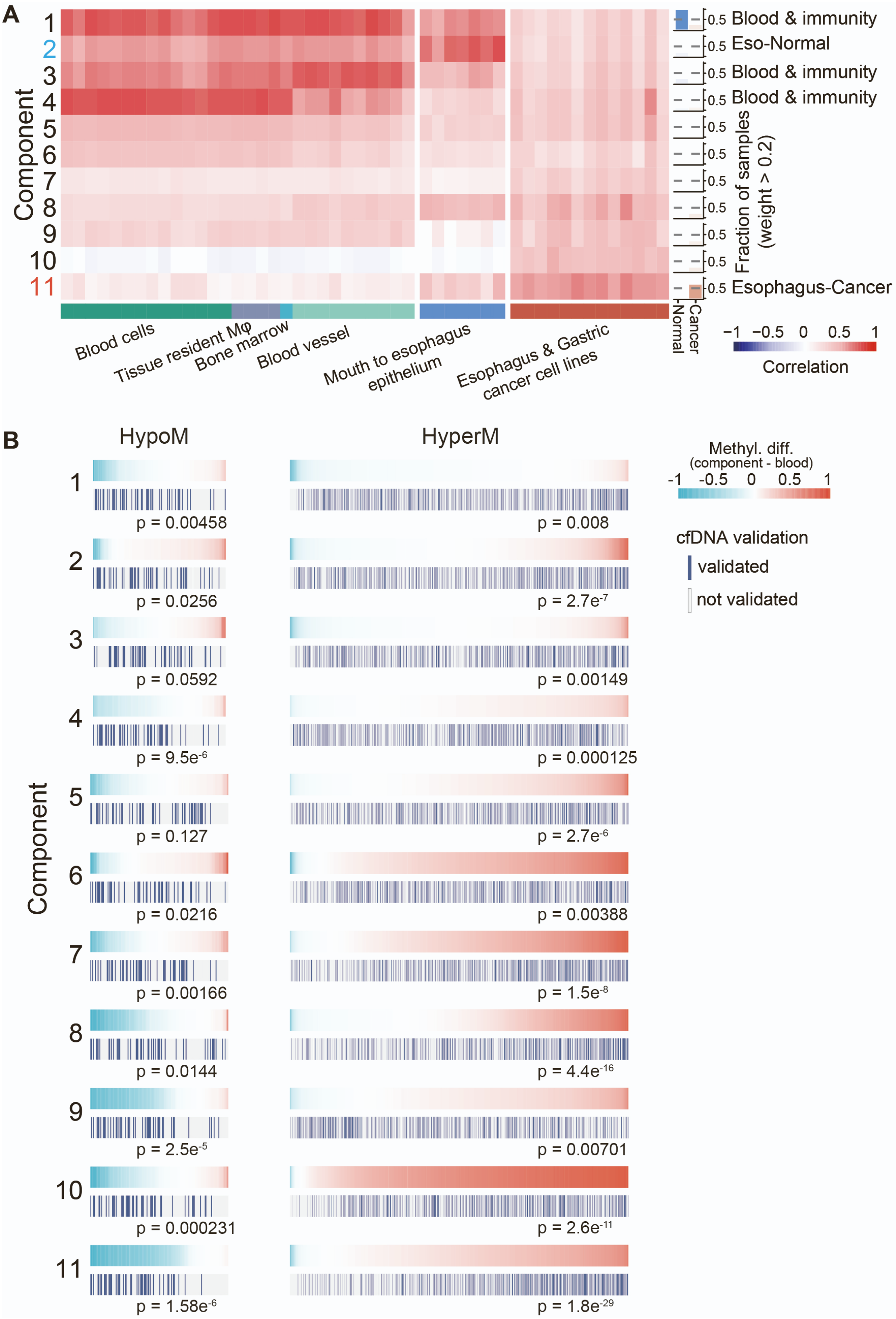

Figure S9. Component determination for esophagus data and validation of tissue cancer markers at the component level using cfDNA samples, Related to Figure 3.

(A) Methylation correlations between various cell types and decomposed esophageal components, alongside the fraction of samples with a component weight > 0.2 (on the right). Each column represents one cell type, and the major types are shown below. The most

strongly associated tissue/cell types of certain components (*e.g.*, component 2 and 11) were identified (blue for normal and red for cancer). The cell types include (from left to right): Blood-B, Blood-B-Mem, Blood-Granulocytes, Blood-NK, Blood-T-CD3, Blood-T-CD4, Blood-T-CenMem-CD4, Blood-T-EffMem-CD4, Blood-T-Naïve-CD4, Blood-T-CD8, Blood-T-Eff-CD8, Blood-T-EffMem-CD8, Blood-T-Naïve-CD8, Blood-Monocytes, Lung-Alveolar-Macrophages, Lung-interstitial-Macrophages, Colon-Macrophages, Liver-Macrophages, Bone marrow-Erythrocyte progenitors, Aorta-Endothelium, Saphenous-Vein-Endothelium, Lung-Alveolar-Endothelium, Liver-Endothelium, Pancreas-Endothelium, Pancreas-Islet-Endothelium, Kidney-Glomerular-Endothelium, Kidney-Tubular-Endothelium, Aorta-Smooth-Muscle, Coronary-Artery-Smooth-Muscle, Larynx-Epithelium, Pharynx-Epithelium, Tongue-Epithelium, Tongue base-Epithelium, Tonsil-Palatine-Epithelium, Esophagus-Epithelium, ECC12, NCI-SNU-16, OCUM-1, T-T, TE-10, TE-15, EMC-BAC-1, EMC-BAC-2, GT3TKB, HCE-4, OE21, RF-48, and SK-GT-2. (**B**) The enrichment of cfDNA-validated pre-defined HypoM and HyperM markers from an existing target methylation sequencing panel for each component. The methylation differences between the spectra of each component and the average of healthy blood cell samples were ranked and displayed (the first row of each component) and the validated markers were annotated (the second row of each component; dark blue labelled). P-values (<0.05) from a Kolmogorov–Smirnov test are provided for each component to indicate the statistical significance.

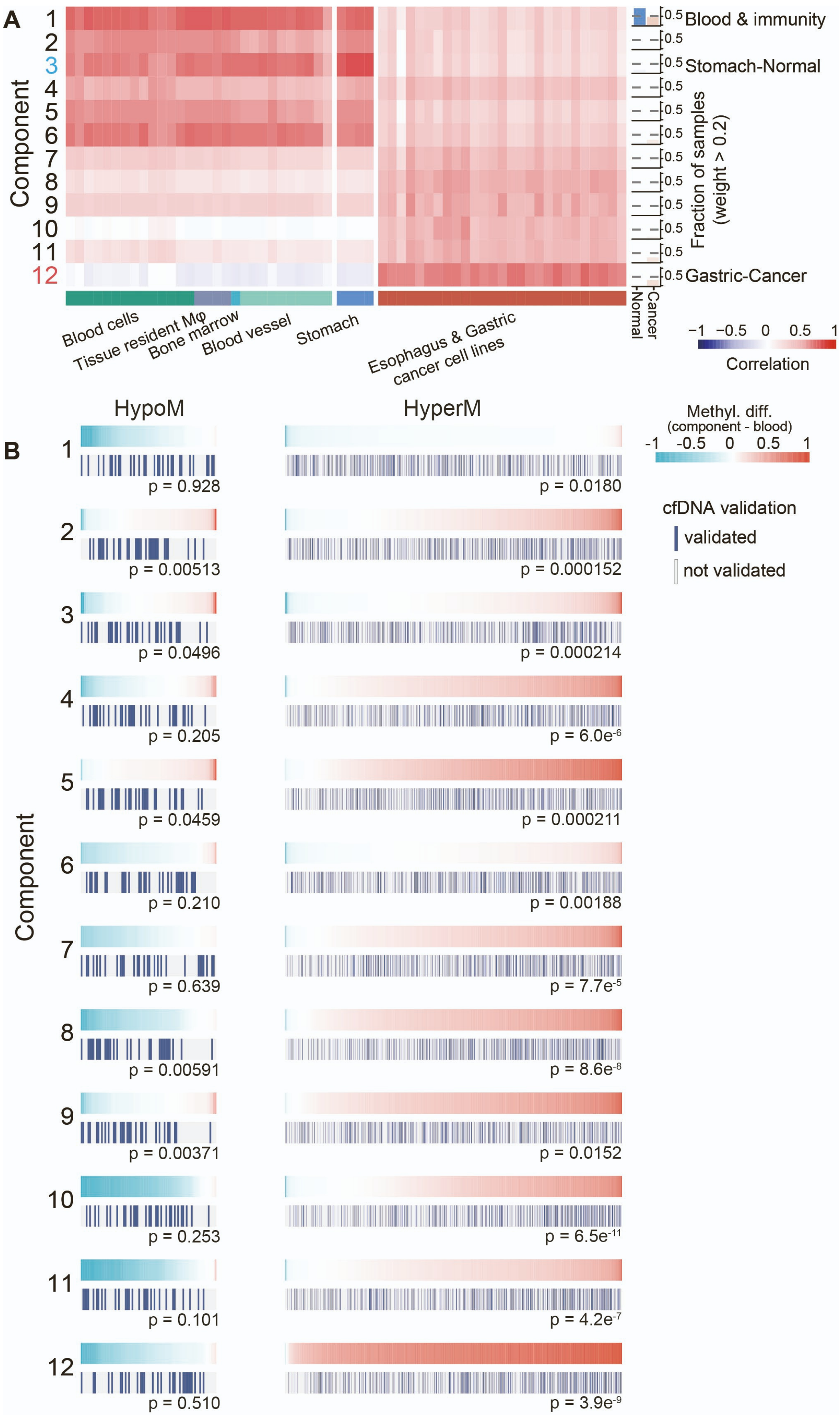

Figure S10. Component determination for gastric data and validation of tissue cancer markers at the component level using cfDNA samples, Related to Figure 3.

The same descriptions for A and B as in Figure S9, but for gastric cancer. The cell types for the gastric tissue comparisons include (from left to right): Blood-B, Blood-B-Mem, Blood-Granulocytes, Blood-NK, Blood-T-CD3, Blood-T-CD4, Blood-T-CenMem-CD4, Blood-T-EffMem-CD4, Blood-T-Naïve-CD4, Blood-T-CD8, Blood-T-Eff-CD8, Blood-T-EffMem-CD8, Blood-T-Naïve-CD8, Blood-Monocytes, Lung-Alveolar-Macrophages, Lung-interstitial-Macrophages, Colon-Macrophages, Liver-Macrophages, Bone marrow-Erythrocyte progenitors, Aorta-Endothelium, Saphenous-Vein-Endothelium, Lung-Alveolar-Endothelium, Liver-Endothelium, Pancreas-Endothelium, Pancreas-Islet-Endothelium, Kidney-Glomerular-Endothelium, Kidney-Tubular-Endothelium, Aorta-Smooth-Muscle, Coronary-Artery-Smooth-Muscle, Gastric-antrum-Endocrine, Gastric-antrum-Epithelium, Gastric-body-Epithelium, Gastric-fundus-Epithelium, AGS, ECC10, KATOIII, MKN45, MKN7, NCI-SNU-1, NCI-SNU-16, NCI-SNU-5, NUGC-4, OE19, KYSE-150, KYSE-410, KYSE-510, TE-1, ESO26, ESO51, HSC-39, KYSE-220, MKN28, NCI-N87, OACM5-1,OE33, SK-GT-2, SK-GT-4, TE-12, and TGBC11TKB.

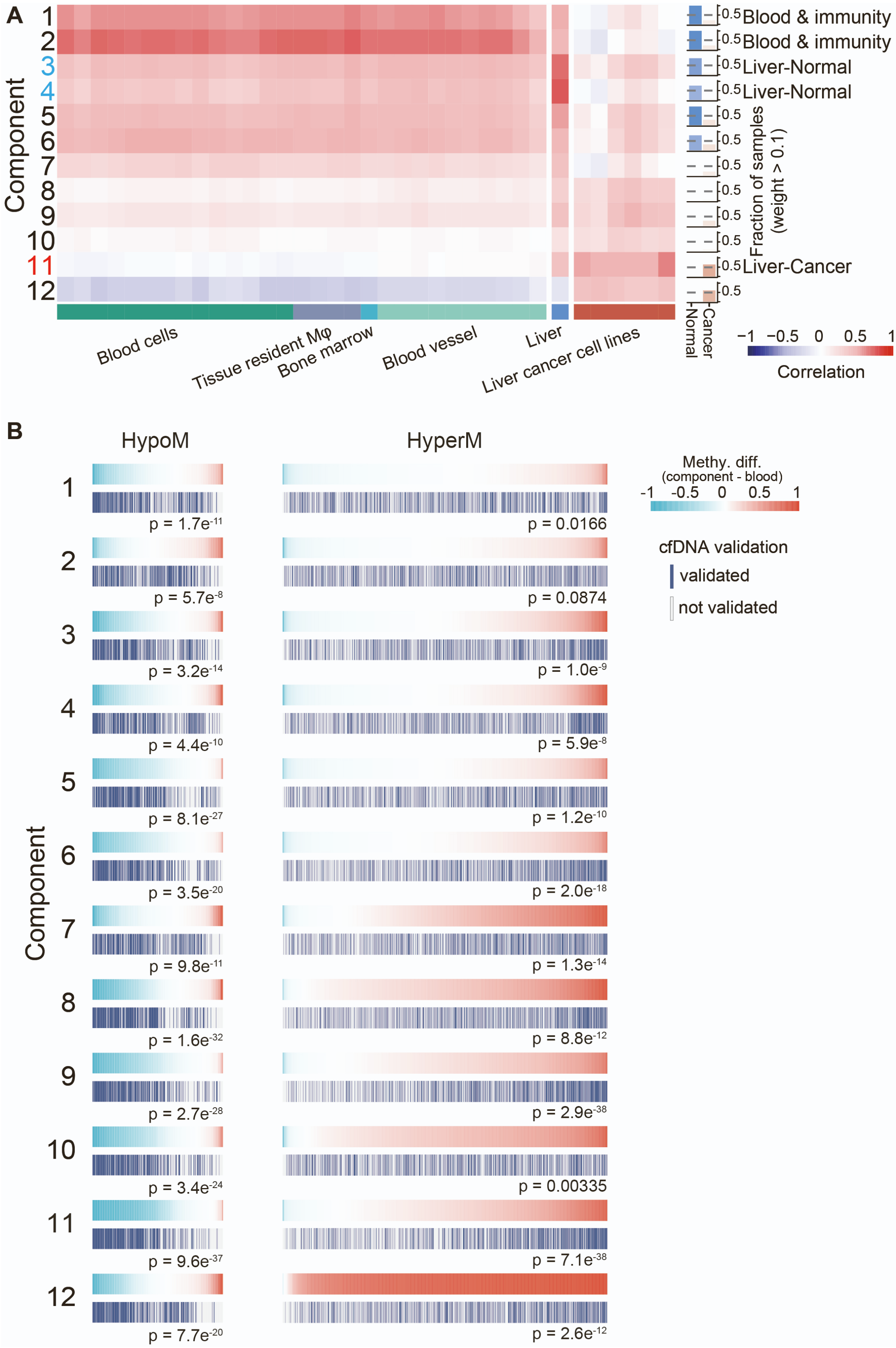

Figure S11. Component determination for liver data and validation of tissue cancer markers at the component level using cfDNA samples, Related to Figure 3.

The same descriptions for A and B as in Figure S9, but for liver cancer. The cell types include (from left to right): Blood-B, Blood-B-Mem, Blood-Granulocytes, Blood-NK, Blood-T-CD3,

Blood-T-CD4, Blood-T-CenMem-CD4, Blood-T-EffMem-CD4, Blood-T-Naïve-CD4, Blood-T-CD8, Blood-T-Eff-CD8, Blood-T-EffMem-CD8, Blood-T-Naïve-CD8, Blood-Monocytes, Lung-Alveolar-Macrophages, Lung-interstitial-Macrophages, Colon-Macrophages, Liver-Macrophages, Bone marrow-Erythrocyte progenitors, Aorta-Endothelium, Saphenous-Vein-Endothelium, Lung-Alveolar-Endothelium, Liver-Endothelium, Pancreas-Endothelium, Pancreas-Islet-Endothelium, Kidney-Glomerular-Endothelium, Kidney-Tubular-Endothelium, Aorta-Smooth-Muscle, Coronary-Artery-Smooth-Muscle, Liver-Hepatocytes, JHH-2, JHH-4, SNU-387, SNU-423, SNU-475, and HUH-1.

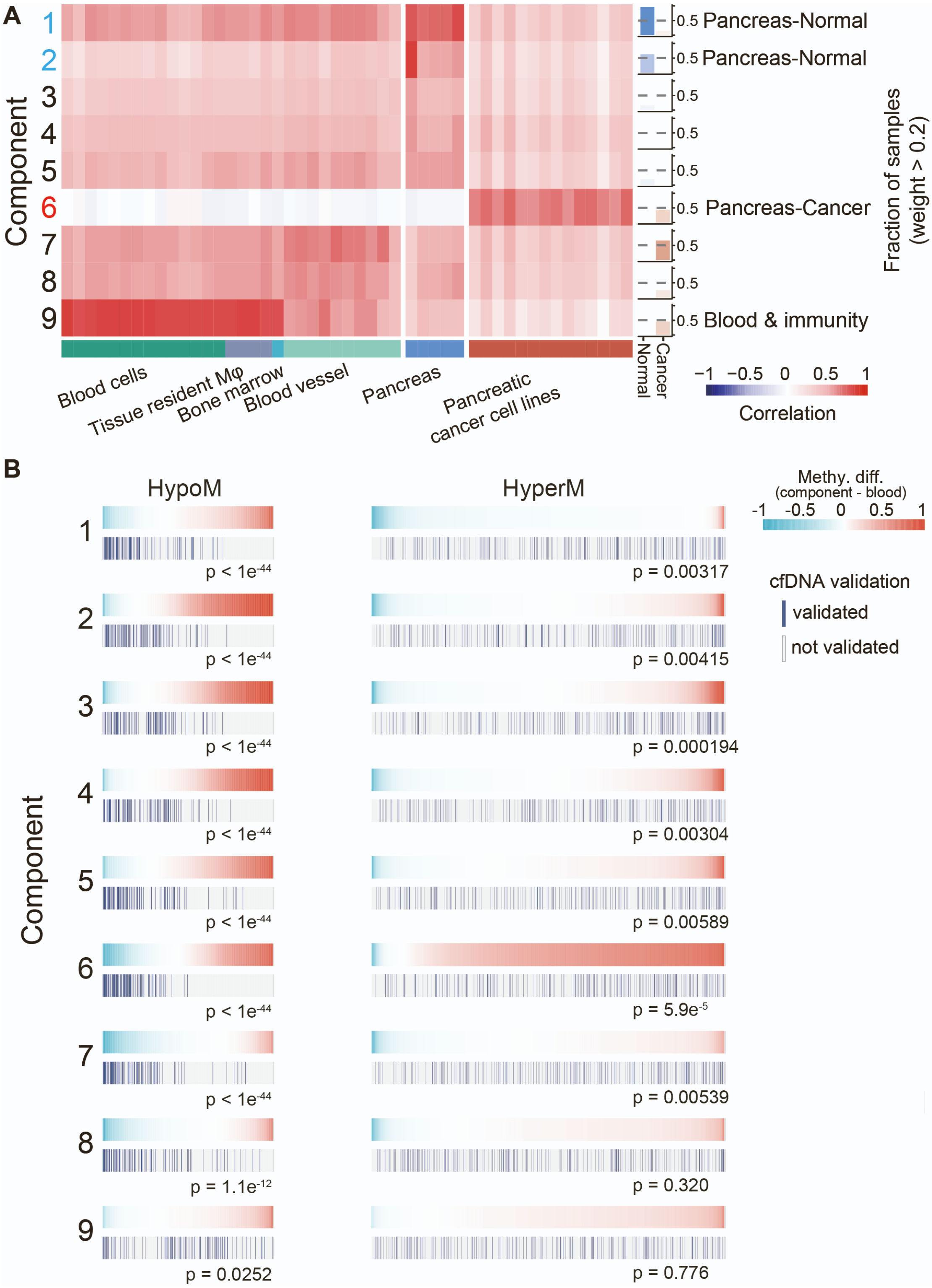

Figure S12. Component determination for pancreas data and validation of tissue cancer markers at the component level using cfDNA samples, Related to Figure 3.

The same descriptions for A and B as in Figure S9, but for pancreatic cancer. The cell types include (from left to right): Blood-B, Blood-B-Mem, Blood-Granulocytes, Blood-NK, Blood-T-CD3, Blood-T-CD4, Blood-T-CenMem-CD4, Blood-T-EffMem-CD4, Blood-T-Naïve-CD4, Blood-T-CD8, Blood-T-Eff-CD8, Blood-T-EffMem-CD8, Blood-T-Naïve-CD8, Blood-Monocytes, Lung-Alveolar-Macrophages,

Lung-interstitial-Macrophages, Colon-Macrophages, Liver-Macrophages, Bone marrow-Erythrocyte progenitors, Aorta-Endothelium, Saphenous-Vein-Endothelium, Lung-Alveolar-Endothelium, Liver-Endothelium, Pancreas-Endothelium, Pancreas-Islet-Endothelium, Kidney-Glomerular-Endothelium, Kidney-Tubular-Endothelium, Aorta-Smooth-Muscle, Coronary-Artery-Smooth-Muscle, Pancreas-Acinar, Pancreas-Alpha, Pancreas-Beta, Pancreas-Delta, Pancreas-Duct, CAPAN-1, CFPAC-1, HPAF-II, PANC-03-27, PANC-10-05, PSN1, SU8686, SW1990, DAN-G, MZ1-PC, PANC-02-03, PANC-04-03, PL4, and YAPC.

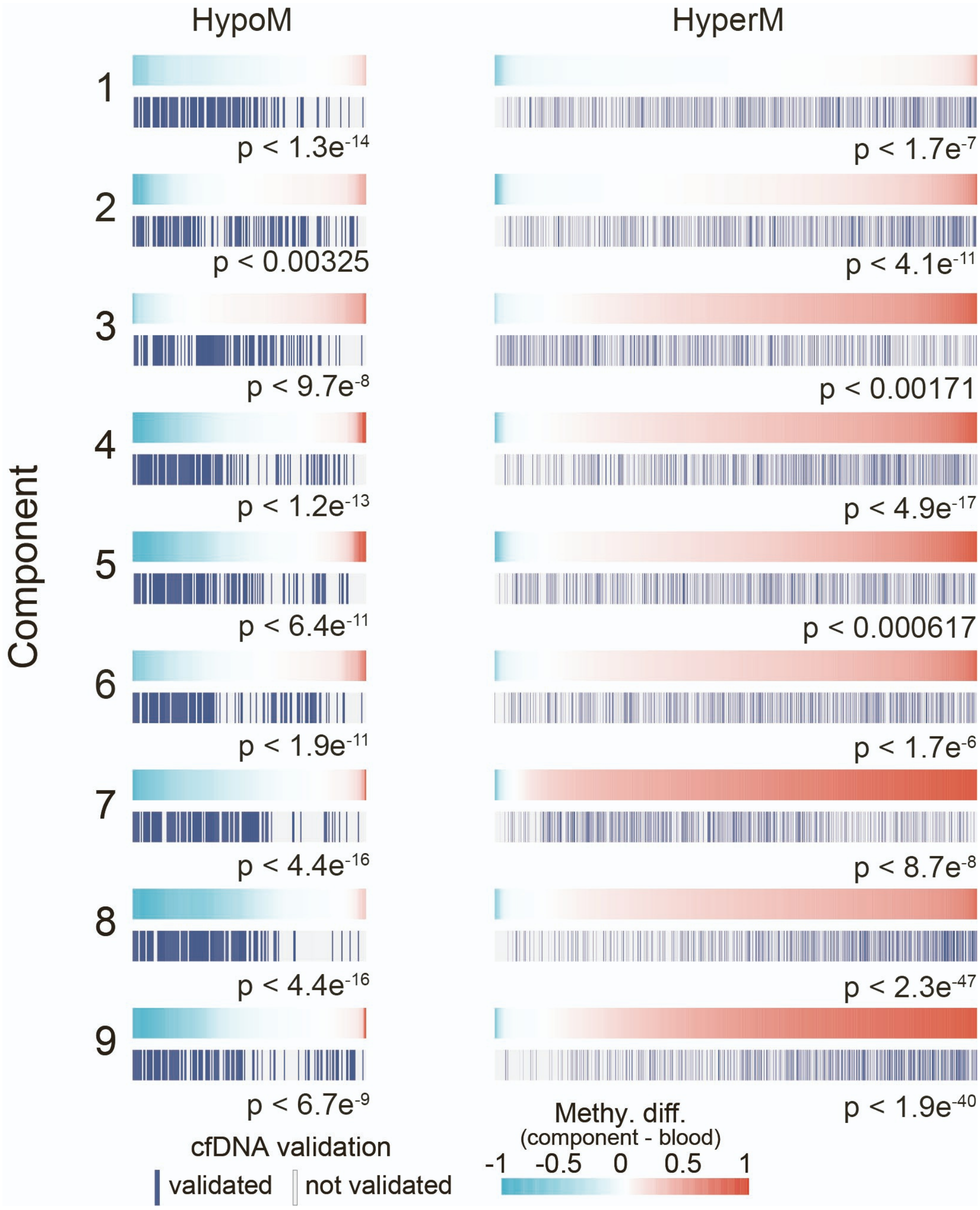

Figure S13. Validation of colorectal tissue cancer markers at the component level using cfDNA samples, Related to Figure 3.

The enrichment of cfDNA-validated pre-defined HypoM and HyperM markers from an existing target methylation sequencing panel for each component. The methylation differences between spectra for each component and healthy blood cells were ranked and displayed. P-values (<0.05) from a Kolmogorov-Smirnov test are included to indicate statistical significance.

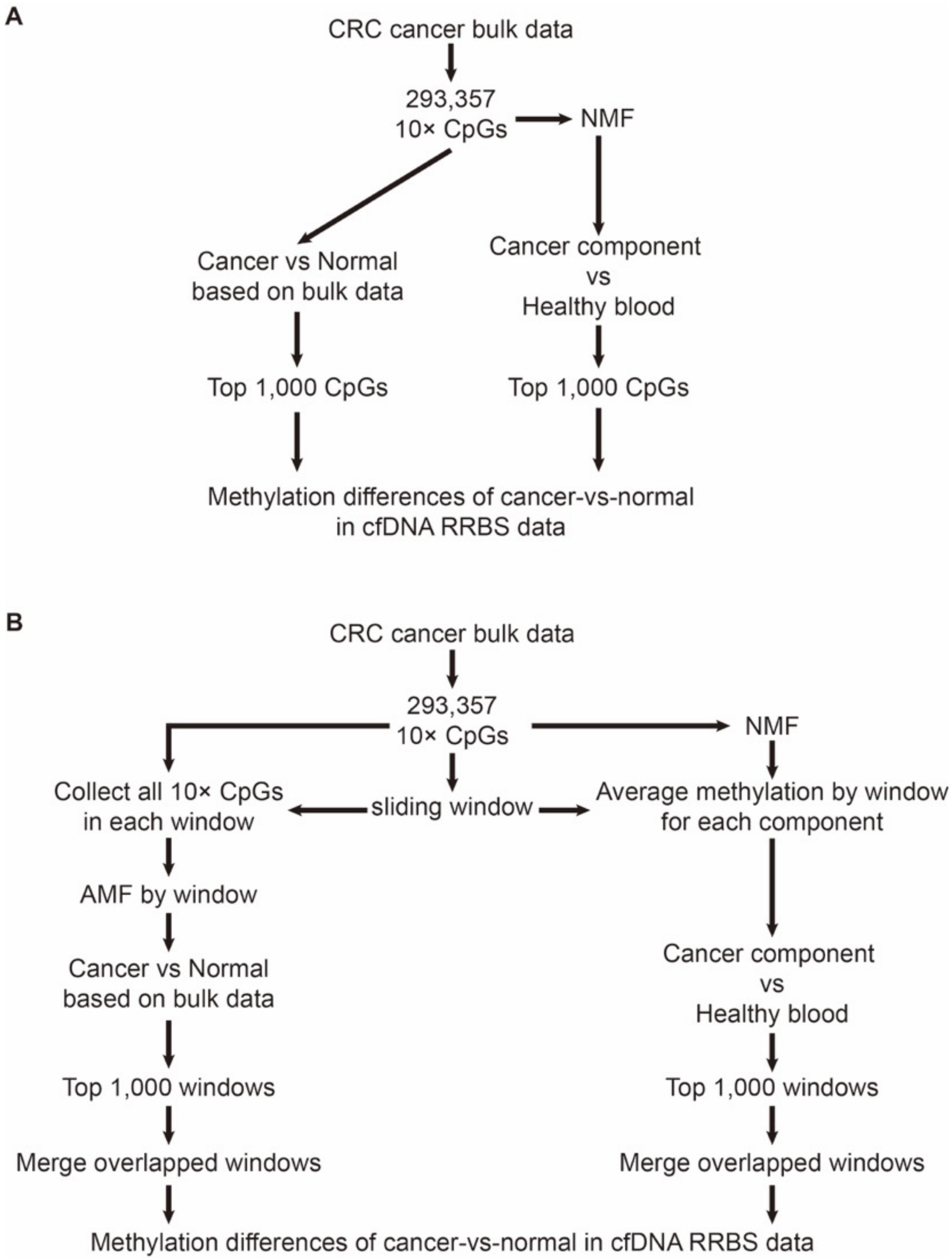

Figure S14. A head-to-head comparison of markers selected through conventional approaches and the decomposition-based framework, Related to Figure 4.

The pipeline to detect the top DMCs **(A)** and DMRs **(B)** via the decomposition-based framework and conventional approaches, which were evaluated in an independent cfDNA RRBS dataset.

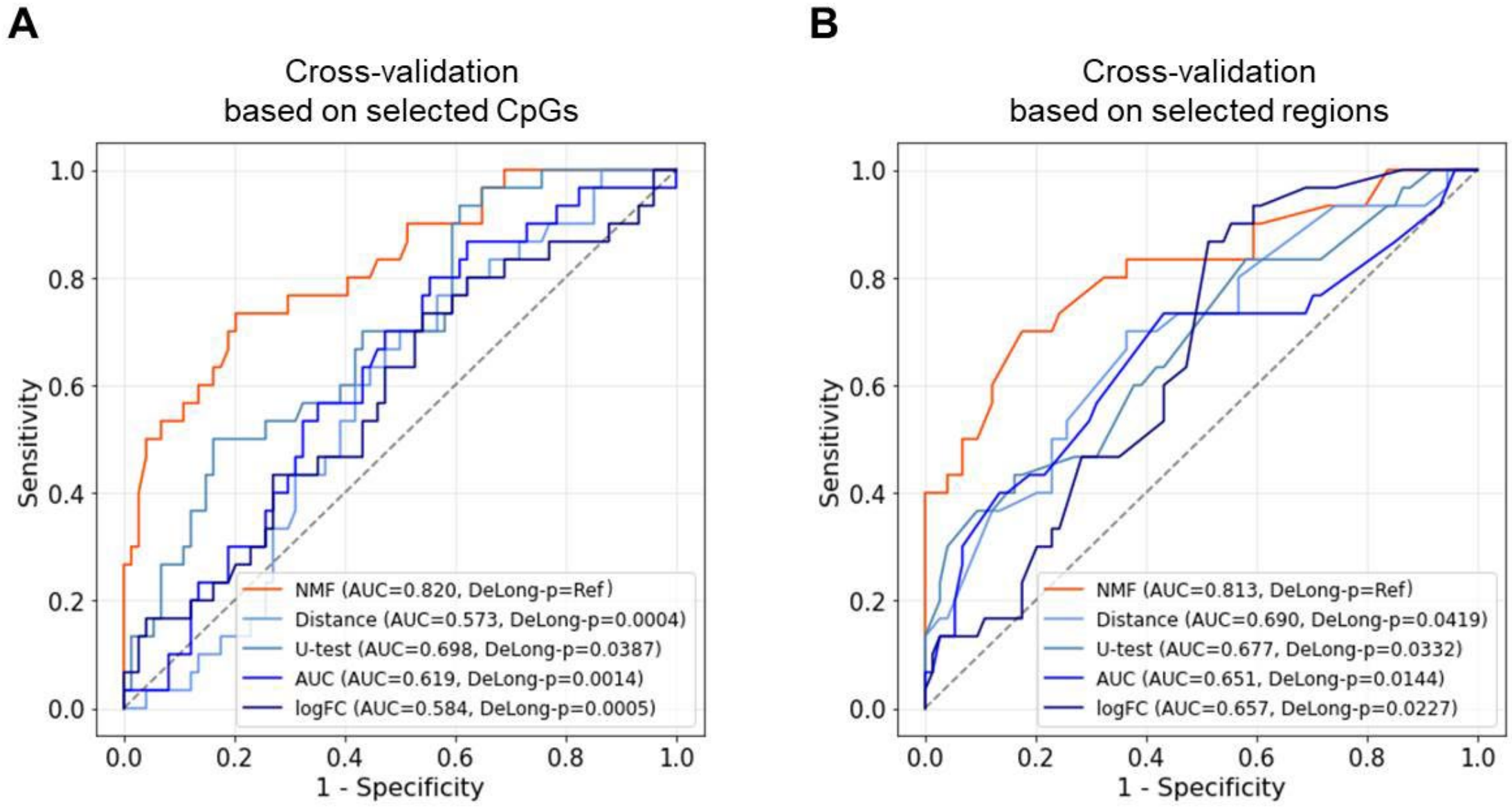

Figure S15. Comparison of cancer prediction accuracy between NMF-based markers and conventionally selected markers, Related to Figure 4.

A three-fold cross-validation was performed using an independent cfDNA RRBS dataset comprising 30 colorectal cancer samples and 74 healthy controls. The top differentially methylated CpGs (A) and regions (B) were selected as features for random forest model construction. Model performance was evaluated by the area under the receiver operating characteristic curve (AUC), and statistical differences between NMF-based predictions and those based on conventional marker selection approaches were assessed using the DeLong test.

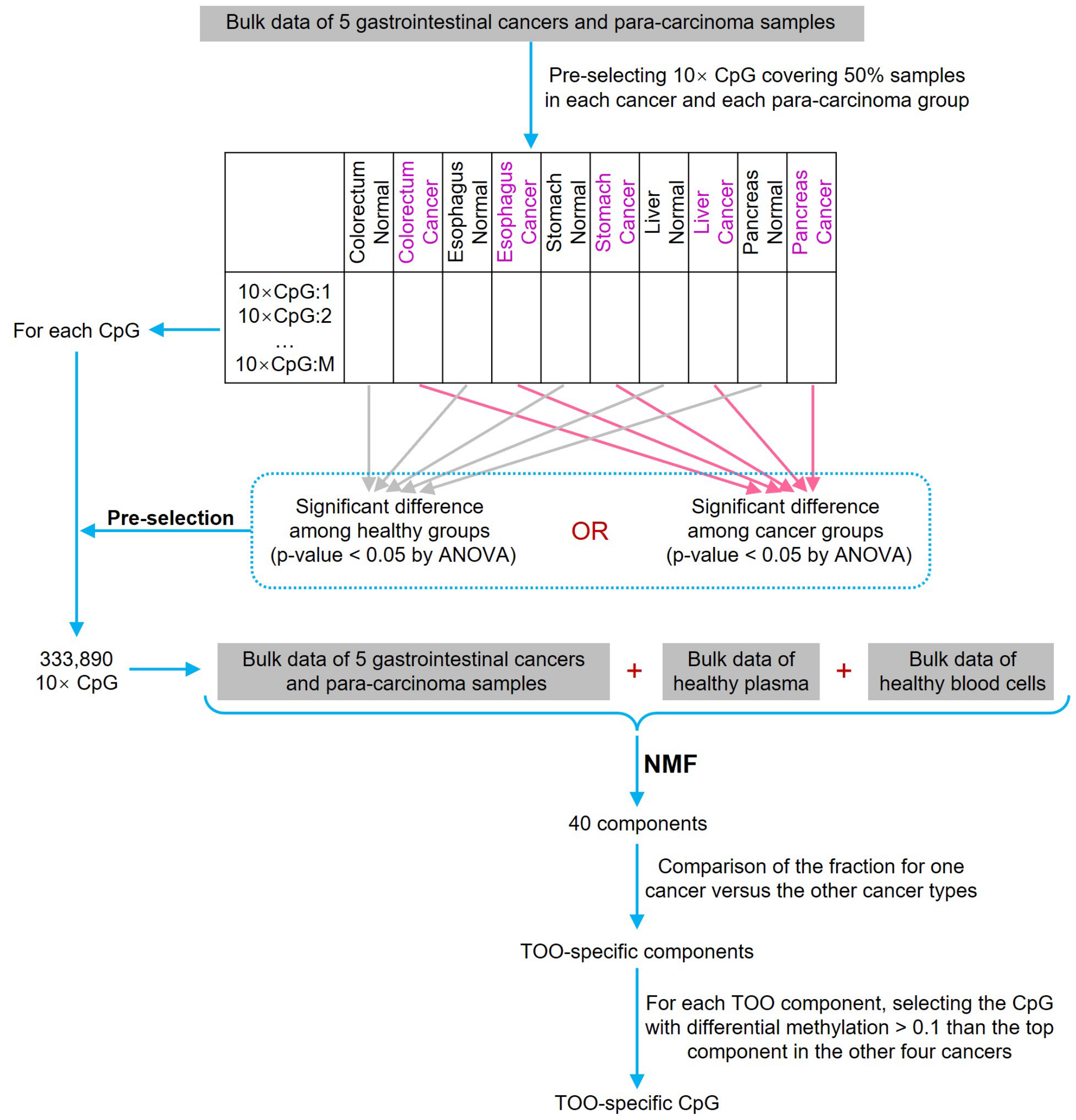

Figure S16. Pipeline to call TOO markers through data decomposition, Related to Figure 4.

The pipeline to call TOO markers based on data decomposition.

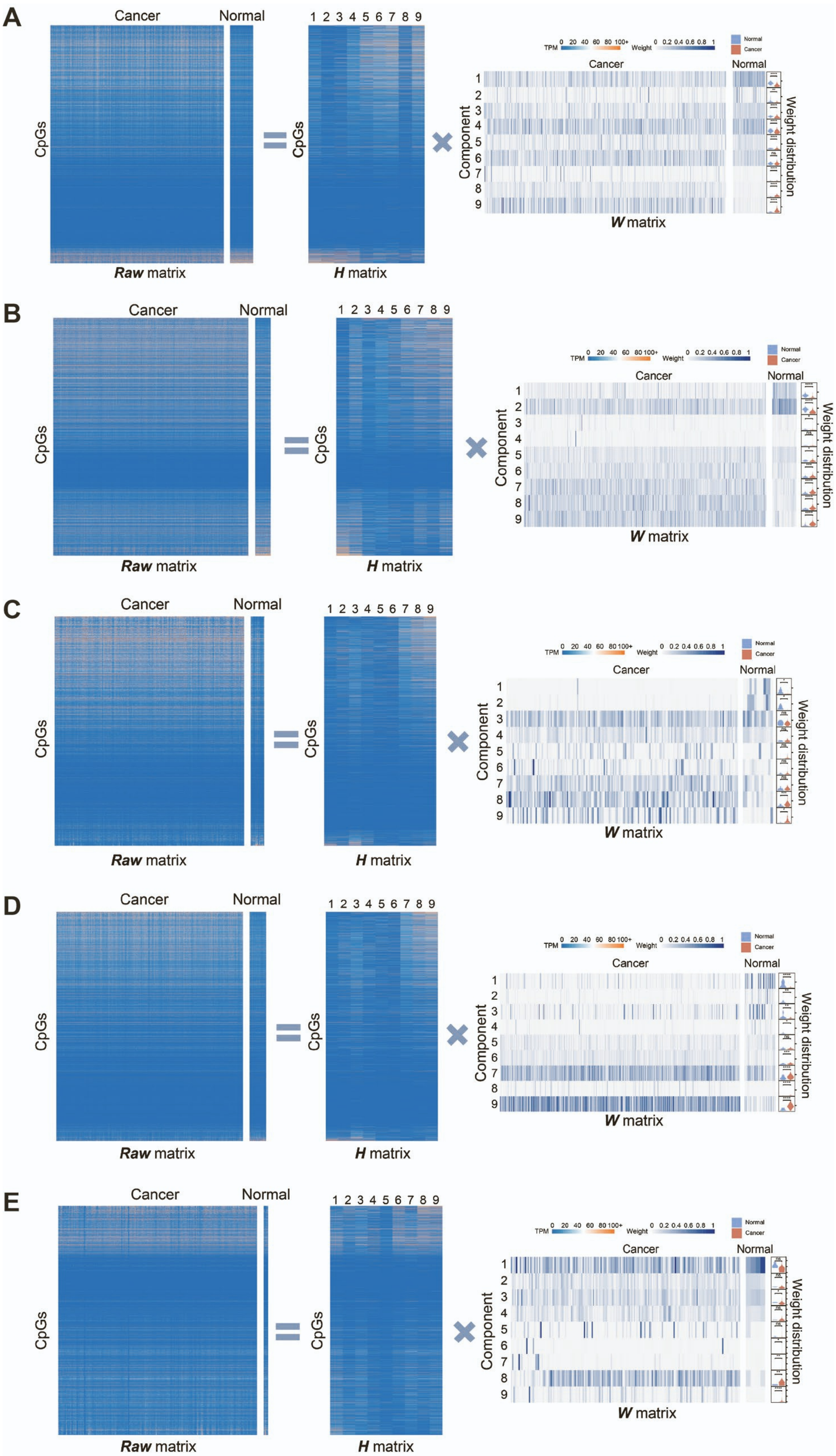

Figure S17. Decomposition of bulk transcriptomic data for five gastrointestinal cancers, Related to Figure 5.

The NMF-based decomposition of RNA-seq data of liver cancer (A), colorectal cancer (B), esophageal cancer (C), gastric cancer (D) and pancreatic cancer (E), with the corresponding para-carcinoma samples, respectively. More details see description in Figure S1.

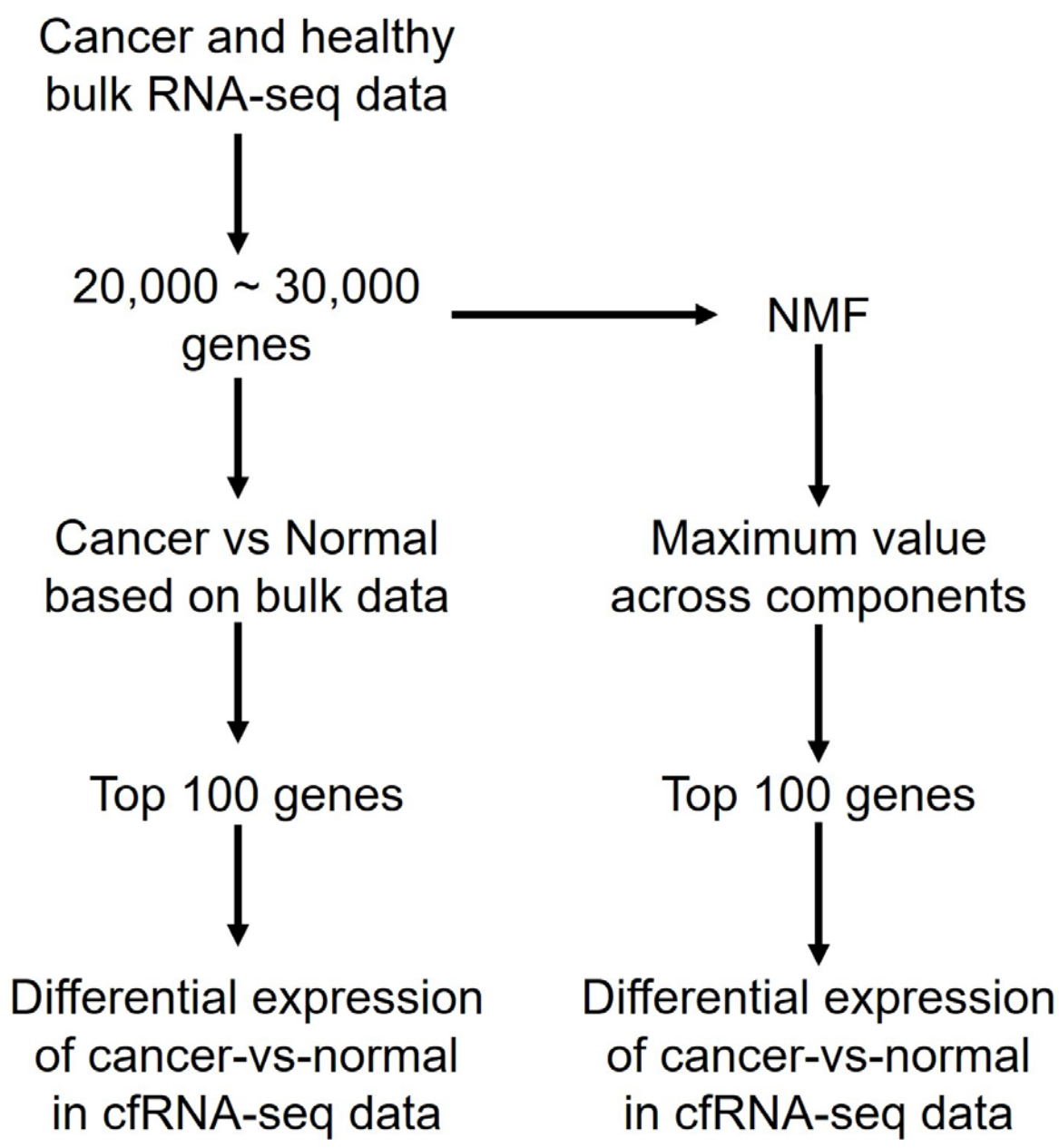

Figure S18. A head-to-head comparison of RNA markers selected through conventional approaches and the decomposition-based framework, Related to Figure 5.

The pipeline to detect the top RNA markers via the decomposition-based framework and conventional approaches, which were evaluated in an independent cfRNA-seq dataset.

Supplemental Tables

Table S1. Validation of the TOO markers selected via data decomposition using a target cfDNA methylation sequencing panel , Related to Figure 4.

TOO-specific component	HyperM DMC					HypoM DMC				
	All DMC	DMC in panel	cfDNA validated DMC	Odds ratio	p-value	All DMC	DMC in panel	cfDNA validated DMC	Odds ratio	p-value
**Colorectum**										
Component 9	8682	42	27	29.256	1.96E-23	1854	98	38	10.335	7.67E-22
Component 10	950	144	99	37.001	4.77E-87	3740	108	42	10.402	4.76E-24
Component 11	4223	195	114	23.823	3.57E-88	4015	134	44	7.994	1.14E-21
Component 12	8445	336	154	14.571	6.68E-98	3230	68	41	24.839	3.10E-33
**Esophagus**										
Component 15	12986	14	12	58.385	3.33E-11	3467	92	15	1.893	0.0233
Component 16	5806	120	51	7.262	8.33E-22	3390	29	2	0.718	0.768
Component 17	1108	53	43	42.222	3.27E-35	5131	59	15	3.316	0.000263
**Stomach**										
Component 18	14261	12	10	36.540	3.38E-08	1362	28	0	0	1
Component 19	4123	14	14	Inf	1.35E-13	5795	51	0	0	1
**Liver**										
Component 21	5528	204	106	11.011	3.79E-55	15998	178	11	0.650	0.943
Component 22	26732	291	144	10.076	7.18E-71	10119	55	6	1.211	0.394
Component 23	18968	293	156	11.761	8.97E-83	6683	58	3	0.539	0.911
Component 24	3367	176	87	9.895	3.27E-43	27094	318	23	0.769	0.909
Component 25	8152	205	107	11.118	6.49E-56	28419	272	24	0.956	0.613
**Pancreas**										
Component 27	1627	18	2	1.555	0.392	3825	218	31	2.074	0.000392
Component 28	15497	116	7	0.798	0.769	1791	21	13	20.302	2.41E-10

Note: Odds ratios and p-values were calculated by a hyper-geometric test based on (1) all 36,102 CpGs contained in the targeted cfDNA methylation sequencing panel, (2) the number of CpGs in the cfDNA TOO markers in the panel for each cancer type (2,117 for colorectal, 3,375 for esophageal, 4,354 for gastric, 3,317 for liver, and 2,687 for pancreatic cancer), (3) the number of decomposition-based TOO-specific DMC covered by the panel (the column "DMC in panel" in the table), and (4) the number of decomposition-based TOO-specific DMC having available cfDNA methylation signals (overlap of (2) and (3); the column "cfDNA validated DMC" in the table).
